# Organic farming enhances soil microbial abundance and activity—A meta-analysis and meta-regression

**DOI:** 10.1371/journal.pone.0180442

**Published:** 2017-07-12

**Authors:** Martina Lori, Sarah Symnaczik, Paul Mäder, Gerlinde De Deyn, Andreas Gattinger

**Affiliations:** 1 Department of Soil Sciences, Research Institute of Organic Agriculture (FiBL), Frick, Switzerland; 2 Karl-Glöckner-Str. 21 C, Justus-Liebig University Giessen, Giessen, Germany; 3 Department of Soil Quality, Wageningen University, Wageningen, The Netherlands; USDA Agricultural Research Service, UNITED STATES

## Abstract

Population growth and climate change challenge our food and farming systems and provide arguments for an increased intensification of agriculture. A promising option is eco-functional intensification through organic farming, an approach based on using and enhancing internal natural resources and processes to secure and improve agricultural productivity, while minimizing negative environmental impacts. In this concept an active soil microbiota plays an important role for various soil based ecosystem services such as nutrient cycling, erosion control and pest and disease regulation. Several studies have reported a positive effect of organic farming on soil health and quality including microbial community traits. However, so far no systematic quantification of whether organic farming systems comprise larger and more active soil microbial communities compared to conventional farming systems was performed on a global scale. Therefore, we conducted a meta-analysis on current literature to quantify possible differences in key indicators for soil microbial abundance and activity in organic and conventional cropping systems. All together we integrated data from 56 mainly peer-reviewed papers into our analysis, including 149 pairwise comparisons originating from different climatic zones and experimental duration ranging from 3 to more than 100 years. Overall, we found that organic systems had 32% to 84% greater microbial biomass carbon, microbial biomass nitrogen, total phospholipid fatty-acids, and dehydrogenase, urease and protease activities than conventional systems. Exclusively the metabolic quotient as an indicator for stresses on microbial communities remained unaffected by the farming systems. Categorical subgroup analysis revealed that crop rotation, the inclusion of legumes in the crop rotation and organic inputs are important farming practices affecting soil microbial community size and activity. Furthermore, we show that differences in microbial size and activity between organic and conventional farming systems vary as a function of land use (arable, orchards, and grassland), plant life cycle (annual and perennial) and climatic zone. In summary, this study shows that overall organic farming enhances total microbial abundance and activity in agricultural soils on a global scale.

## Introduction

Microbial communities play an important role in agricultural systems due to their involvement in many different soil processes and functions. In soils, microbial communities are the engines driving nutrient transformation and release, as well as being directly and indirectly involved in many other ecosystem services such as erosion control by formation of stable soil aggregates and soil structuring [1,2] or pest and disease regulation [3]. Besides many other biological, chemical and physical traits, a fertile soil harbours an abundant, active, diverse and adaptive microbial community which underlies soil functioning [4–6].

There is a great need for agricultural systems that are capable of producing enough food while coping with changing climatic conditions, but which do not further increase the exploitation and degradation of Earth’s limited resources [7]. A possible option is eco-functional intensification through organic farming, an approach based on making optimal use of internal natural resources and processes to secure and improve agricultural productivity, while minimizing negative environmental impacts such as loss of biodiversity, nutrient leakage and soil degradation. Organic farming is defined by national but also broader regulations such as the European Union guideline *Council Regulation (EC) No 834/2007 on organic production and labelling of organic products and repealing Regulation (EEC) No 2092/91* or global guidelines such as IFOAM regulation [8]. Nowadays, approximately one per cent of the world’s farmland is organically managed. Since 1999, a growth of more than 300% has been noted. North America has the biggest retail sales of organically farmed products though harbours less than 7% of the total worldwide organic production area scaled [8].

In organic farming, agricultural production aims at closing nutrient cycles, in which plant residues or manure from livestock are returned back to the fields, at the same time incorporating perennial and leguminous plants. In addition, neither synthetic fertilizers nor synthetic pesticides are applicable. This concept heavily relies on an active microbial community to break down organic matter into plant available nutrients. Conventionally managed systems as well profit from a high abundance of microorganism considering their involvement in nutrient retention [9] and soil structure improvement [1] which might positively influence nutrient efficiency but also water dynamics. Recovery of N in crop plants is usually less than 50% worldwide [10].

Different measures can be applied to analyse microbial communities, especially microbial abundance and activity are well known indicators for soil quality. Most recent approaches make use of molecular methods such as shotgun or amplicon sequencing to measure diversity and quantitative polymerase chain reaction (qPCR) to measure abundance or activity but many more exist. Unfortunately, there are so far only few studies making use of the same molecular method for comparing microbial communities in organic and conventional farming systems. Indeed, to investigate differences in size and activity of microbial communities, most studies rely on traditional methods. Microbial biomass carbon and nitrogen are indicators used to assess the soil microbial community size and are measured using the chloroform fumigation method [11]. Phospholipid fatty-acids (PLFA) biomarkers are often used to identify certain microbial groups and community patterns, but the total amount of PLFAs also refer to the abundance of microbial communities [12–14]. To assess soil microbial activity ‘dehydrogenase’, the ‘basal respiration’ and ‘metabolic quotient’ serve as widely used indicators. Dehydrogenase activity reflects intracellular microbial redox processes, thus functioning as an indicator for soil microbial metabolic activities [15]. The metabolic quotient describes the amount of basal respiration, the steady state respiration in the soil by microorganisms, per unit microbial biomass carbon [16], allowing its use as an indicator for stress or disturbance as microorganisms divert more energy from growth into maintenance [17]. Hence a high metabolic quotient reflects a microbial community under stress.

Available nitrogen is often the most limiting plant nutrient in agricultural systems. The invention of the Haber-Bosch process, where dinitrogen reacts under high pressure and temperatures with hydrogen, forming urea [18], had and still has a huge impact on agriculture with a drastic increase of human driven reactive nitrogen in the global nitrogen cycle during the last decades. Currently, most conventional agricultural fertilisation practices heavily rely on synthetic mineral nitrogen sources. Mineral nitrogen fertilizer tend to have an enhanced risk of nitrogen losses, especially via leaching and run-off, compared to organic nitrogen fertilizer, which harbour a relatively small fraction of its nitrogen in mineral form. Furthermore, there are indications that mineral fertilization negatively impacts soil microbiota [19], whereas organic nitrogen fertilization improves biological activity and soil C and N content [20,21]. Anyhow, both fertilization strategies can be used with or without harmful effects to nature, depending on their use and handling. Nevertheless, the highly energy-consuming process of synthetic mineral nitrogen production and its direct and indirect harm to the environment and the fact that natural fossil resources are finite, have to be considered when planning agricultural expansion in future.

In order to avoid dependencies on synthetic nitrogen fertilizers, organically bound nitrogen in form of manures, slurries and other organic residues is introduced into the system instead of synthetic fertilizers. Though to sustain plant production under organic fertilization, soils inevitably need a high capacity to break down organically bound nitrogen into minerals, which then become available to plants. Extracellular microbial enzymes such as proteases are involved in the initial step of breaking proteins down into peptides, dipeptides and single amino acids [15,22], whereas ureases break down urea into carbon dioxide and ammonia [15], which make these two enzymes potential indicators for soil microbial mineralization capacity.

Over the last twenty-five years several individual scientific studies comparing microbial communities’ abundance and activities of organically and conventionally managed systems have been performed and published. However, comprehensive syntheses and analyses of the current knowledge are still rare and not up to date as the last review about long-term effects of organic farming on microbial parameters is twenty-five years old [23].

In this work, a systematic literature study followed by a meta-analytical approach aims to quantify possible differences in key indicators for soil microbial abundance and activity between organic and conventional agricultural systems on a global scale.

Following our own experience from the well-known DOK farming systems trial [24–26] and some anecdotal evidence from other studies and long-term trials [27–29], we hypothesized that i) overall microbial abundance ii) overall microbial activity and iii) overall mineralisation potential is enhanced in organic compared to conventional systems. Furthermore, categorical meta-analyses were used for the identification of farming practices displaying a positive influence on microbial community size and activity. Finally, the possible effect of farming system-induced factors on microbial size and activity was identified with a meta-regression approach.

## Materials and methods

### 1. Data source and selection criteria

To quantify the effect of long-term organic farming on total abundance and activity of soil microbial communities, results from 56 studies and 149 paired comparisons were analysed in a meta-analysis (S1 Table). In order to find pairwise comparisons of organic and conventional farming systems, a comprehensive literature search with the terms ‘organic microb’, ‘organic farming microb’, ‘farming system microb’, ‘agriculure microb’ and ‘microbial community farming’ was conducted in online search engines google scholar and web of science starting in June 2015 and ending in February 2016. Furthermore, reference lists of identified studies were manually browsed to find additional articles not identified using search terms. Peer-reviewed scientific journals as well as grey literature were considered, as long as minimal experimental standards were met. Papers were considered eligible and included in the meta-analysis if the following criteria were met (S1 Fig):

1. organic systems are defined as such by the authors and organic practices need to be applied for at least three consecutive years prior to sampling. The term conventional summarizes farming practices relying on synthetic nitrogen fertilizer and chemical plant protection wherein also more modern integrative approaches, e.g. use of organic inputs and integrated plant protection, are included.
2. farming system comparison needs to be pairwise, meaning the compared fields are subjected to the same pedo-climatic conditions (e.g. temperature, precipitation, soil type)
3. minimal experimental standards are required, meaning a sample size (n) of at least three independent replicates along with the reporting of standard deviation (SD), standard error (SE) or significance level
4. only data deriving from open field and greenhouses were considered. Experimental incubation studies in the lab or pot trials were not included since they do not reflect *in situ* soil conditions properly

The following criteria for data handling were applied after studies were selected.

1. if several fertilization doses were applied, always ‘full fertilization’ often referred to as ‘recommended’ was chosen. Furthermore, if several fertilizer treatments were compared within the same study, they were handled as individual comparisons
2. when several studies from a certain site exist or results have been published twice in different contexts and journals, the latest sampling or the study with the most complete dataset, respectively, was included except when the reported data comprised different target variables
3. when different crop rotations from a certain site existed, they were handled as individual comparisons

For each study, the following target variables along with their SD or SE and n of each treatment were extracted: Microbial biomass carbon, microbial biomass nitrogen and total PLFA, representing size of the microbial community. Basal respiration, dehydrogenase activity and the metabolic quotient, which was in approximately 50% of studies calculated via given CO_2_-C production rate per unit microbial biomass carbon, representing microbial community activity. Urease and protease activity are indicators for microbial communities’ nitrogen mineralization capacity. For a few studies, supplementary data from the authors was required and obtained via personal email correspondence. When data were only given graphically, the online tool webplotdigitzer (http://arohatgi.info/WebPlotDigitizer/) was used for more standardized data extraction. Additional information on several study characteristics, pedo-climatic conditions and farming practices were collected as covariates for further categorical analysis and meta-regression. About half of the studies did not indicate climatic zone of the study site and thus a high resolution map was used to identify the missing data [30].

### 2. Data analysis

As target variable outcomes are measured on a physical scale and are unlikely to be zero, the response ratios (RR) of organic and conventional microbial parameters were used to identify differences between the two farming systems. RRs were calculated as the ratio between the mean (X) of organic and conventional target variables.

$$RR=\frac{X_{org}}{X_{con}}.$$

All computations were carried out as followed in log scale to obtain normally distributed data using *Ln*_*RR*_. The variance of the RR (V_lnRR_) was calculated via

$$\;V_{\text{ln}RR}=S^{2}{}_{pool}*\left(\frac{1}{\left(n_{org}*X_{org}{}^{2}\right)}\right)+\left(\frac{1}{\left(n_{con}*X_{con}{}^{2}\right)}\right)$$

[31],

where S_pooled_ is the pooled sample standard deviation and n the number of replicates.

A random effects model (95% confidence interval) weighing each individual observation according to its precision based on n and SD using inverse variance (IV) was used [32]. In cases where only SE was given, SD was calculated via $\text{SE}\sqrt{n}$. Some studies reported neither SD nor SE for certain target variables, in which case SD was calculated individually for each comparison using the average coefficient of variation based on the corresponding dataset [33]. However, within all eight target variables, SD or SE were reported in the respective study or provided via personal communication with the authors in 60% up to 90% of all cases. In order to test whether overall effect sizes statistically differ from RR = 1 and therefore show statistically significant effect, Z-statistics were used. The Z-statistics is a widely used method for combining results from several independent investigations in order to test the same overall hypothesis [32,34]. Heterogeneity was estimated using the I^2^ statistics, which calculates the proportion of total variance due to between study variance and hence measures the variability between studies on a scale from 0 to 100% [35]. All datasets showed a rather high I^2^ ranging from 54% to 96% (S2 Table), indicating heterogeneity deriving from true between study variance. To explore possible drivers for the observed differences, such as management practices or pedo-climatic factors, further analyses were required and hence random effects categorical meta-analyses were performed. The more categories a subgroup analysis includes, the higher is the chance for a type 1 error to occur. In order to obtain a broad overview, rather many categories were selected and thus the occurrence of type 1 errors needs to be considered when interpreting the results. The following categories were selected and analysed: Köppen climatic zone (A-D), continents, land use (arable, grassland, orchard), plant coverage (annual/perennial), clay content (low, moderate, high), time since conversion (3 years, 4–10 years, >10 years,), crop rotation (similar/different), legumes in crop rotation (no legumes = no; legumes in both systems = ORG+CON; legumes only in the organic system = ORG; and legumes only in the conventional system = CON), organic inputs applied (no organic inputs = no; organic inputs in both systems = ORG+CON; organic inputs only in the organic system = ORG; organic inputs only in the conventional system = CON), synthetic pesticides used (only in conventional system = CON; no synthetic pesticides in both systems = no), and set up (long-term experiment or farm comparison). Categorical meta-analysis was applied to detect differences between farming systems in certain categories. Whenever less than three comparisons per grouping remained, the respective category was excluded from further analysis, indicated as “no data” in the results table. Results for categories with only few comparisons, although statistically significant, should be handled very cautiously and further research filling these knowledge gaps is highly recommended. For target variables showing an overall effect which significantly differs from RR = 1, a categorical effect is present when confidence intervals (CIs) overlap with RR = 1. For the target variable ‘metabolic quotient’, which does not show a significant overall effect, the opposite is the case: a categorical effect is present whenever the CIs do not overlap with RR = 1.

Meta-regression was computed by method of moments using a random effects model with a 95% confidence interval and Knapp and Hartung adjustments [32,36] built on soil organic carbon (SOC), total soil nitrogen (TN) and pH. The farming system induced factors SOC, TN and pH consist of two values for each comparison, one from the organic system and one from the conventional. In order to overcome this problem, the difference and the mean of those paired covariates were calculated and used in the model. We did not include further covariates in the model since we would risk losing too many studies due to incomplete datasets. Furthermore, the other recorded covariates have already been tested in categorical meta-analysis.

All computational procedures were performed in the software comprehensive meta-analysis version 3 (Englewood, NJ: Biostat. www.comprehensive.com). Forest plots were made using SigmaPlot version 11.0 (Systat Software, Inc., San Jose California USA, www.systatsoftware.com).

### 3. Publication bias

In order to assure quality control, each individual study was carefully analysed for minimal experimental standard; if these standards were not met, the study was excluded. To assess publication bias, the RR of each comparison was plotted against the SE, which results in a funnel plot. A symmetrical dispersion of these data points indicates that no bias exists. Duvall and Tweedie’s trim and fill, a non-parametric, rank based augmentation technique, was applied to detect and fill funnel plot asymmetry with hypothetical values [37,38].

### 4. Non-independent data

Each study on a certain microbial target variable contained at least one comparison of one organic and one conventional farming system per site. However, several studies reported data from more than one comparison per site such as one organic system compared to two different conventional systems with different fertilizer regimes or crop rotations. When integrating all these data, this situation leads to non-independent data and might bias the results via narrow confidence intervals that lead to overestimating significant results. One option to avoid this problem is to aggregate data in a synthetic effect size, wherein out of many dependent comparisons, only one comparison is made. A negative aspect of aggregating effect sizes is the loss of single comparisons, which strongly affects feasibility of categorical meta-analyses. For this reason and with the knowledge that our overall meta-analysis displayed very strong results, minimizing the risk of overestimating significant results, this approach was dismissed as it was also dismissed in previous studies by Gattinger et al. (2012) [39], Skinner et al. (2014) [40], Seufert et al. (2012) [41] and many more. However, results of categorical meta-analyses have to be handled more cautiously due to lower study numbers per group compared to overall meta-analysis.

## Results

### 1. General data evaluation

**Fig 1:**
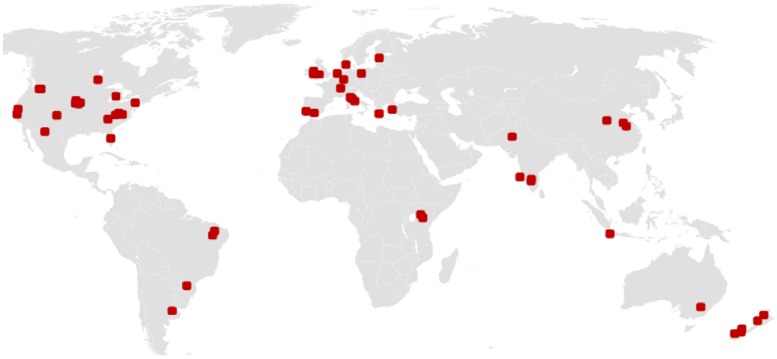
Origin of the study sites included in the meta-analysis. Each dot represents a site included in the meta-analysis.

In total, literature search yielded 56 eligible independent studies including 149 pairwise comparisons (experimental field trials and farm comparisons) originating from all continents, except Antarctica, and spanning the Köppen climatic zones A to D (Fig 1, Table 1, S1 Table). 55 studies originated from peer-reviewed scientific journals, whereas only one non-peer-reviewed study derived from grey literature and only one study is yet unpublished but complied with all criteria for minimal scientific standard. Organic systems were managed 16.1 years on average since conversion from conventional and soil sampling was up to twenty centimetres depth but identical in all paired studies. In the vast majority of studies, soils were tilled in organic and conventional sites though some studies as well compared the effect of reduced or no-till between the farming systems. In a few comparison tillage differed between organic and conventional but only to a small extent.

**Table 1:** Overview of the obtained studies and paired comparisons, target variables and main study characteristics.

	Total	Microbial biomass carbon	Microbial biomass nitrogen	Total PLFA	Basal respiration	Dehydrogenase activity	Metabolic quotient	Protease activity	Urease activity
**Number of studies**	56 studies	42 studies	21 studies	8 studies	29 studies	17 studies	22 studies	3 studies	7 studies
**Number of paired comparisons**	149	100	49	22	66	40	40	7	18
**Experimental field comparisons**	75	54	28	11	42	18	26	2	5
**Farm comparisons**	74	46	21	11	24	22	14	5	13
**Experimental duration in years (Mean / Median)**	16.1 / 10.0	17.2 / 10.0	11.0 / 10.0	25.9 / 10.0	12.6 / 10.0	8.8 / 5.5	12.3 / 8.0	6.6 / 6.0	7.9 / 7.0
**Coverage of climatic zones**^+^	A, B, C, D	A, B, C, D	A, B, C, D	A, C, D	A, B, C, D	A, B, C, D	A, B, C, D	C	A, B, C
**Coverage of continents** *	6 of 6	6 of 6	5 of 6	4 of 6	6 of 6	5 of 6	6 of 6	3 of 6	4 of 6

* all except Antarctica

^+^ A = Tropical/mega thermal climates, B = Dry climates, C = Temperate/mesothermal climates, D = Continental/Microthermal climates, E = Polar climates

Funnel plot analysis followed by Duval and Tweedies’s trim and fill test [37,38] detected a major publication bias in basal respiration, which subsequently got excluded from any further analysis. The target variables microbial biomass carbon, microbial biomass nitrogen and metabolic quotient displayed a minor, negligent publication bias each, which did not lead to a change in significance level when adjusted accordingly. All other target variables did not show any sign of publication bias (S3 Table). However, in small datasets assessments of funnel plot asymmetry are quite likely underpowered [42] and thus results have to be handled more carefully.

### 2. Differences among farming systems

**Fig 2:**
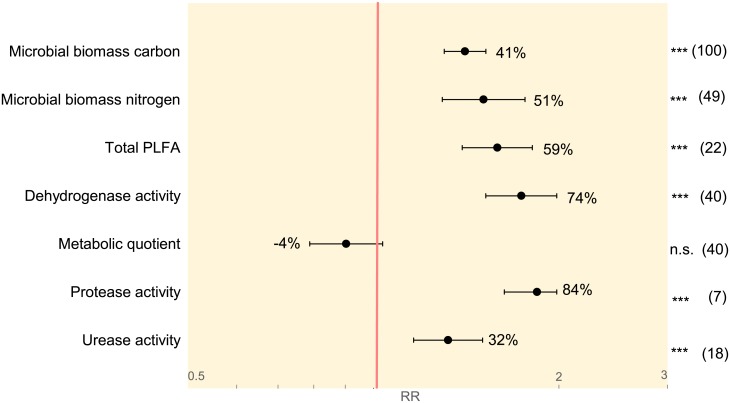
Summary of overall response ratios (RR). Random effects model with a Z-Distribution and a 95% confidence interval was applied on seven target variables listed on the y-axis. The red line (RR = 1) indicates no difference between organic and conventional systems. X-axis is given in log-scale as indicated by grey numbers. Numbers in parentheses display the number of pairwise comparisons included in each calculation. Numbers beside the confidence intervals indicate the overall percentage difference per target variable. *≤0.05, **≤0.01, ***≤0.001, n.s. = not significant.

The main objective of the present study was to identify whether significant differences in microbial community abundance and activity in soils under organic and conventional farming management exist on a global scale. Six out of seven investigated target variables were significantly increased in organic compared to conventional farming systems; only the metabolic quotient of the soil microbes was not statistically different between the two farming systems (Fig 2, S2 Table). The following results are presented in parentheses as “RR, lower CI to upper CI, p-value”.

Microbial biomass carbon (1.41, 1.30 to 1.52, p <.0001), microbial biomass nitrogen (1.51, 1.29 to 1.76, p <.0001) and total PLFA (1.59, 1.39 to 1.81, p<0.0001) were on average 41%, 51% and 59% higher, respectively, in organically than conventionally farmed soils. Dehydrogenase activity was on average 74% (1.74, 1.52 to 1.98, p<0.0001) greater under organic management, whereas the metabolic quotient displayed a reduction of 4% by trend in organic compared to conventional farming systems(0.96, 0.84 to 1.09, p<0.05). Protease and urease activity were increased by 84% (1.84, 1.63 to 1.98, p<0.0001) and 32% (1.32, 1.16 to 1.50, p<0.0001) respectively, in organic compared to conventional systems.

In summary, meta-analysis revealed that organic farming exerts a strong positive overall effect on the abundance and activity of soil microbial communities in agricultural systems at a global scale. Furthermore, the results of all target variables showed a high heterogeneity (I^2^) ranging from 54% to 96% (S2 Table). Heterogeneity and not homogeneity is the standard in meta-analyses and has to be considered when choosing the appropriate model [43]. Due to heterogeneity, random effects model, which is able to account for between-study variability, was chosen over fixed effects model. Furthermore, high heterogeneity allows exploring possible drivers, which might be associated with the between-study variability, such as differences in climatic conditions, farming practices and their induced changes in soil abiotic factors. Therefore, we performed further investigations using categorical meta-analyses and meta-regression to verify the association of pedo-climatic, land management and soil abiotic factors with soil microbial community abundance and activity in relation to organic vs conventional soil management.

### 3. Factors influencing soil microbial communities

#### 3.1 Pedo-climatic factors

**Fig 3:**
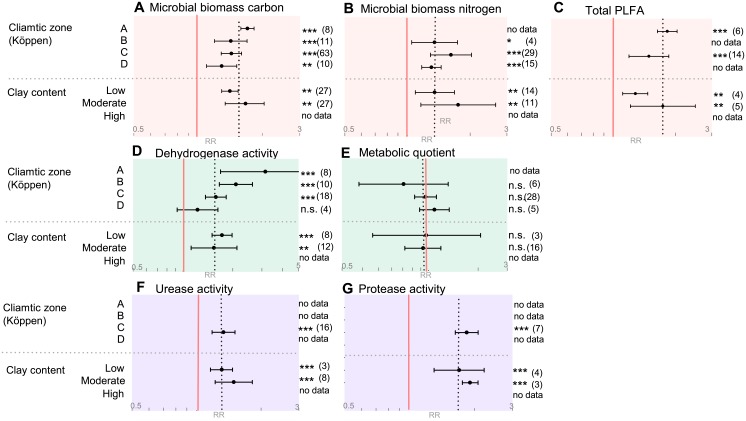
Summary of pedo-climatic categories for all target variables as affected by organic vs conventional farming systems. Categorical random effects model (Knapp-Hartung) with a 95% confidence interval was applied. A response ratio (RR) of 1.0, marked with a red line, indicates no difference between organic and conventional systems. The overall RRs for each target variable are indicated by the vertical black dotted lines. X-axis is given in log-scale as indicated with grey numbers. ‘No data’ indicates that less than three comparisons for a respective category were available and hence no analysis was performed. Numbers in parentheses indicate the number of pairwise comparisons included in each calculation. Different categories are listed on the y-axis. Köppen climatic zones are abbreviated as followed: A = Tropical/megathermal climates, B = Dry climates, C = Temperate/Mesothermal climates, D = Continental/Microthermal climates. Plots A-C summarize all target variables representing microbial size, D+E represent target variables for microbial activity and F+G represent target variables involved in nitrogen mineralization activity. Significance levels: *≤0.05, **≤0.01, ***≤0.001, n.s. = non-significant.

The increase in microbial biomass in response to organic soil management was apparent in all climatic zones and the effect sizes did not significantly differ between climatic zones (Fig 3A–3C, S4 Table). In contrast, the effect of organic soil management on microbial activity differed between climatic zones (Fig 2D). Dehydrogenase was up to three times (3.15, 1.69 to 5.89, p<0.001) more active in organic compared to conventional systems in climatic zone A (tropical/megathermal), while no difference between the farming systems could be identified in climatic zone D (continental/microthermal) (1.22, 0.92 to 1.63, p>0.05) (Fig 3D, S4 Table). For the target variables urease and protease activity only data from climatic zone C (temperate/mesothermal) are available, hence no statements about putative climatic influences on microbial mineralisation activities of organic nitrogen via these enzymes can be drawn (Fig 3F and 3G). Overall, the vast majority of studies set originates from climatic zone C, whereas climatic zones A and B are highly underrepresented.

No influence of soil clay content on differences between organic versus conventional soil management was identified for any target variable (Fig 3A–3G, S4 Table).

#### 3.2 Management factors

**Fig 4:**
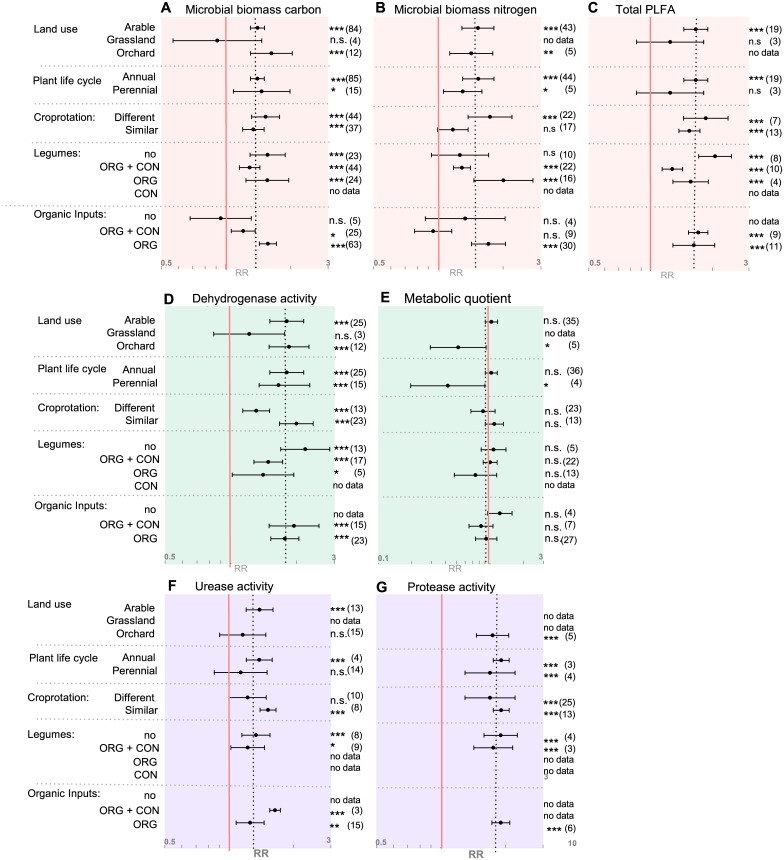
Summary of management categories for all target variables as affected by organic vs conventional farming systems. Categorical random effects model (Knapp-Hartung) with a 95% confidence interval was applied. A response ratio (RR) of 1.0, marked with a red line, indicates no difference between organic and conventional systems. The overall RRs per target variable are indicated by the vertical black dotted lines. X-axis is given in log-scale as indicated with grey numbers. ‘No data’ indicates that less than three comparisons for a respective category were available and hence no analysis was performed. Numbers in parentheses indicate the number of pairwise comparisons included in each calculation. Different categories are listed on the y-axis. No = ‘no legumes’/‘no organic input’ in both farming systems. ORG = organic, CON = conventional/non-organic systems. Plots A-C summarize all target variables representing microbial size, D+E represent target variables for microbial activity and F+G represent target variables involved in nitrogen mineralization activity. Significance levels: *≤0.05, **≤0.01, ***≤0.001, n.s. = non-significant.

Different agricultural land uses such as arable cropping, orchards or grasslands influence differences in microbial abundance and activity between the two farming systems (Fig 4). Microbial biomass carbon was significantly increased in organic arable cropping (1.4, 1.3 to 1.51, p<0.001) and organic orchards (1.63, 1.30 to 2.04, p<0.001) as compared to the conventional counterparts, whereas no impact of farming system could be detected in grassland (Fig 4A, S4 Table). A similar response to land use was identified for total PLFA where an increase in organic systems occurred exclusively under arable land use (1.66, 1.45 to 1.89, p<0.001) and not in grasslands (Fig 4C, S4 Table). In addition, the response of microbial activity to organic management was influenced by land use, with increased dehydrogenase activity in arable sites and orchards (1.81, 1.51 to 2.16, p<0.001 and 1.85, 1.50 to 2.29, p<0.001), whereas in grasslands organic management did not affect dehydrogenase activity (Fig 4D and 4E, S4 Table). In addition, the metabolic quotient was on average 50% lower in organic compared to conventional orchards (0.52, 0.29 to 0.92, p<0.05), whereas it persisted unaffected in arable sites; no data were available for grassland (Fig 4E, S4 Table). For the target variables urease and protease activity not enough data were available to test effect sizes.

Furthermore, the differentiation between annual and perennial plant life cycle also influenced soil microbial community activity. The metabolic quotient displayed significantly lower values in organic perennial systems compared to conventional perennials (0.42, 0.20 to 0.90, p<0.05), whereas no difference was observable in annual systems (Fig 4E, S4 Table). It has to be noted that even though statistically different from a RR equal one, the metabolic quotient of perennials consists of very few study numbers and wide confidence intervals and thus results have to be taken cautiously.

The ability of a plant to fix nitrogen and thus its chemistry may also influence soil microbial communities. We therefore analysed the impact of the presence of legumes as green manure, cover crop or cash crop in the crop rotation on the effect of organic management on soil microbial biomass and activity. The inclusion or exclusion of legumes affected differences in microbial biomass nitrogen. Whenever both systems lack legumes in their rotation, no difference between organic and conventional was found in microbial biomass nitrogen. In contrast when both systems include legumes, the organic system displayed a higher microbial nitrogen content than the conventional counterpart (1.29, 1.18 to 1.41, p<0.001) (Fig 4B, S4 Table). Further in-depth evaluations regarding the effect of frequency of legumes in the rotation or sampling time point could not be undertaken due to restrictions in the design of the dataset. In cases where exclusively the organic systems contain legumes, the difference in microbial nitrogen between the two system was the most pronounced (2.01, 1.46 to 2.75, p<0.001).

Organic and conventional farming systems often differ in crop rotations in that organic rotations tend to be longer, more diverse and often include leguminous plants. The effect of organic management on soil microbial biomass and activity was largely the same whether or not the crop rotations were similar. However, for microbial biomass nitrogen the effect of organic management disappeared when the rotations were similar (Fig 4B, S4 Table), whereas for dehydrogenase and urease activity the effect of organic management became stronger when the rotations were similar (Fig 4D + 4F, S4 Table).

As no synthetic nitrogen fertilizers are allowed in organic farming, these systems heavily depend on green manure, nitrogen fixation and organic inputs. An active microbial community, which is able to break down organic nitrogen into mineral form, is essential in order to provide plants with adequate levels of nitrogen. In cases where neither organic nor conventional systems received organic inputs, no differences between the two farming systems, especially with regard to microbial biomass carbon, could be observed. Whenever exclusively the organic systems received organic input, microbial biomass carbon increased in response to organic management (Fig 4A, S4 Table).

Categorical subgroup analysis indicates that the average global response towards enhanced microbial community abundance and activity in organically managed system does not hold true for all categories considered. Certain climatic conditions, land use and farming practices indicate to enhance or diminish the positive effect of organic farming, with the largest positive effects being observed in dehydrogenase activity and in response to climatic zone.

#### 3.3 Management induced soil properties

In order to detect farming system induced soil properties and their possible effects on microbial communities, we employed a two-step approach to find potential causative reasons for the observed phenomena. First, the sub set on soil property data encompassing soil organic carbon (SOC), total nitrogen (TN) and pH was subjected to a meta-analysis in order to analyse whether or not organic farming led to an enrichment in the soil (Table 2). Across the entire dataset targeting microbial community traits, SOC was on average 19% (1.19, 1.14 to 1.23, p<0.001), TN 13% (1.13, 1.07 to 1.20, p<0.001) and pH 2% (1.02, 1.002 to 1.04, p<0.01) higher in organic than in conventional systems. Secondly, random effects model meta-regression with the covariates SOC, TN and pH of each system, as the difference and the mean, was performed with six out of seven target variables (S5 Table). In our model, meta-regression displayed solely microbial biomass carbon to be statistically related to SOC (p = 0.007) (S5 Table). The larger the SOC difference between the two farming systems, favouring the organic, the more microbial carbon is found in the organic system. Therefore, we surmise that microbial biomass carbon might be influenced by SOC status of the soil. For microbial biomass nitrogen, no significant soil abiotic driver could be identified. Dehydrogenase activity on the other hand related with pH differences (p = 0.0025, respectively)—the larger the pH difference between the organic and conventional systems and being higher in the organic system, the larger also the response ratio of dehydrogenase activity. A similar pH association was observed for urease activity (p = 0.0187), whereas also a TN association was observable (p = 0.0363). The model did not fit the PLFA and metabolic quotient dataset and hence no explanation for heterogeneity can be drawn in this regard. The protease activity was not included in meta-regression analyses since too few comparisons were available to run the model. Similar to categorical meta-analysis, meta-regression is exclusively an exploratory approach, and its power strongly depends on the amount of comparisons included in the model. Nevertheless, using meta-regression, possible associations could be identified between farming system dependent abiotic factors and microbial indicators. Based on our model we hypothesise that organic farming reduces soil acidification and increases SOC, which beneficially influences microbial community abundance and activity.

**Table 2:** Meta-analysis based on the microbial target variables dataset to asses farming system induced effects on SOC (SOC), total nitrogen (TN) and pH chemistry. The effect of organic and conventional managed systems on SOC, TN and pH was analysed by a random effects model with a 95% confidence interval (CI) and a Z-distribution. RR>1 indicates enhanced values in organic systems.

Target variables	n	RR	Lower 95% CI	Upper 95% CI	Z-value	p-value
SOC	143	1.19	1.14	1.23	11.13	≤0.001
TN	105	1.13	1.07	1.20	7.28	≤0.001
pH	117	1.02	1.00	1.04	2.20	0.002

## Discussion

### 1. Dataset and limitations

Basal respiration displayed major publication bias and adjustments lead to a different significance level (S3 Table). Therefore and with the knowledge that the dehydrogenase activity, which did not show publication bias, also serves as an indicator for microbial activity, basal respiration was excluded from any further analyses. Different factors may serve as explanations for the observed bias. One factor often biasing meta-analyses is the so called ‘file drawer effect’, where studies with no or only marginal significant results get published less often compared to studies with more significant effects [44]. Another explanation for the observed publication bias in the basal respiration dataset might be this methods’ high sensitivity to biotic and abiotic factors during sampling and analysis [45,46]. All other target variables showed no or a rather small publication bias, not leading to a change in significance level when adjusted accordingly, and allowed a good differentiation between the two farming systems. However, even though no publication bias was detected, the results of small datasets such as protease activity, urease activity and also total PLFA have to be handled carefully because, in such cases, tests applied to detect funnel plot asymmetry are usually underpowered [42]. Kicinski and co-workers [47], who conducted the largest meta-study targeting publication bias so far, found that publication bias was smaller in meta-analyses including more recent studies, compared to meta-analyses including older studies. Thus, we can consider this knowledge in our datasets, where tests for publication bias might be underpowered. Hence, in our smallest datset, protease activity, which does not include studies older than 20 years, we can be more confident about unbiased results. Regarding the datasets on microbial biomass carbon and microbial biomass nitrogen, they are unusually large and thus publication bias test result are very likely valid and reliable.

The coverage of geographic location and land use of the pairwise comparisons was unbalanced, being dominated by arable sites in climatic zone C, representing temperate/mesothermal climates with a maximum monthly average above 10°C in their warmest months and above -3°C in their coldest. Areas with scarce soil resources, large-scale land degradation or extreme weather conditions could especially benefit from the more soil building approach that organic farming offers. Data about the performance of organic farming and impact on soil quality including microbial characteristics in these areas are therefore highly needed, especially in the context of global climate change with a projected temperature rise to occur in the temperate zones [48]. In this context the system comparison trials conducted in Kenia, India and Bolivia investigating the effect of organic and conventional farming on several soil parameters are of great value [49–51]. However, more studies are needed to generate comprehensive understanding in arid and semi-arid ecosystems.

### 2. More abundant and more active soil microorganisms in organic farming?

#### 2.1 The effect of farming systems on community size and activity

In 1992 Richard Dick [23] reviewed the effect of long-term organic farming on soil biochemical and microbial parameters while most recently Reeve and colleagues (2016) and Lorenz and Lal (2016) synthesized the effects of organic farming on soil health, food quality and environment [52,53].

However, the current study is the first systematic review quantifying the impact of organic compared to conventional long-term farming on soil microbial community abundance and activity, and is based on twenty years of data collected on a global scale. In this study, a positive effect of organic farming on microbial communities was identified. Six of seven key indicators for microbial community abundance and activity, though to different extents, are significantly enhanced in organic farming systems when compared to conventional systems (Fig 2). Besides the overall activity, indicated by greater dehydrogenase activity, also the protease and urease activity were strongly increased in organic systems. Together this indicates an intensified nitrogen mineralization capacity in organic than in conventional systems.

Unfortunately, our dataset does not enable to test whether enhanced microbial abundance and activity are also correlated with higher plant productivity and more stable yields because yield data were not reported in the vast majority of the included studies. Therefore, direct conclusions cannot be drawn. However, since the effect of farming practice on plant productivity and yields has been compared in previous meta-studies [41,54], it can be assumed that abundance and activity of soil microbial communities does not directly correlate with plant productivity which is on average 20% lower in organic systems [41,54]. In addition, other factors like the choice of crop varieties and agricultural inputs such as chemical fertilizers and pesticides strongly determine yield of farming systems.

In general, we show that overall organic management positively affects microbial pools and activities related to soil carbon and nitrogen cycling.

### 3. Drivers of enhanced soil microbial abundance and activities

As organic farming is a combination of several different farming practices, the use of a general meta-analysis makes it virtually impossible to differentiate between the possible influence of specific management factors and pedo-climatic factors on the observed microbial community differences.

#### 3.1 Pedo-climatic conditions influence microbial activities

To assess a possible influence of climatic conditions on microbial communities, a categorical meta-analysis using Köppen climatic zones A, B, C and D was applied. A categorical analysis with a higher degree of climatic resolution was unfortunately not possible due to the low number of studies per climatic subgroup. No association of climatic zone with observed differences in microbial community abundance was identified, whereas for microbial activity an association with climate zone was detected (Fig 3D). Climatic zone A, also referred to as tropical or mega thermal climate, is characterised by a mean annual temperature of 18°C and a minimum monthly precipitation of 60 mm [30,55]. In climatic zone A, microbial communities in soils under organic management showed up to three times more dehydrogenase activity than those under conventional management. This difference declined gradually over climatic zones B and C to D, where no difference between the two farming systems was observed any longer (Fig 3D). Climatic zone D, also known as continental/microthermal climate, is characterised by average temperatures above 10°C and below -3°C in the warmest and coldest months, respectively [30,55]. It is well known that low temperature hinders fast mineralisation of SOC and freshly added organic matter, and thus generally high SOC levels result in similar microbial activity due to temperature sensitivity of the microbial communities.

The low resolution of climatic zones might be a reason why the remaining target variables did not respond to climatic zones. Nevertheless, differences in microbial activity between organic and conventional systems were shown to some extend being dependent on climate and are most likely linked to differences in temperature, though other factors such as precipitation might also be involved.

As global climate change prognoses indicate strong alterations in polar and temperate regions [48], the beneficial effect of organic farming might rise in areas where the difference is not as pronounced yet. Twenty years ago, Lotter et al. [56] identified that organically farmed plots at the Rodale Institute Farming System had significantly higher yields during severe drought conditions when compared to conventional plots (1984–1989), which also occurred in the following hurricane driven rainy year of 1999 [56]. Organic farming was shown to improve soil physical and chemical properties such as soil organic matter content [26,39,57], aggregate stability [58], higher polysaccharide content and thicker top soil [59]. These properties likely support better water infiltration and storage and thus might be advantageous considering the climate changed induced rainfall variability.

We recommend conducting more primary research on the performance and soil microbial communities of organic and conventional systems outside climatic zones C and D.

#### 3.2 Management factors influence microbial communities

The absence or presence of organic amendments in the form of liquid and solid farmyard manure or green manure crops influenced differences in microbial biomass carbon, whereas it did not significantly affect differences in microbial activity (Fig 4D–4G). Additionally, the meta-regression identified a positive overall association between SOC and microbial biomass carbon (S5 Table). Our results are in line with previous studies reporting similar findings of organic inputs to enhance SOC and microbial biomass carbon [39,60–62]. An interesting aspect not taken into account in the present study is how different kinds of organic input can affect the observed differences in microbial abundance and activity. Tu and his co-workers (2005) identified that the quality and quantity of different inputs, such as composted cotton gin trash, animal manure and green manure, could impact microbial abundance and activity in organic tomato production differently [61]. Our dataset did not allow for more in-depth investigation in this regard since literature search criteria were not designed accordingly. Thus, we recommend a detailed systematic analysis in order to assess the effect of quality, quantity and timing of organic matter application on microbial activity and abundance.

A long and diverse crop rotation combined with the inclusion of legumes helps mitigate weed, insect and pathogen pressure, while also enhancing crop yields [63–66]. On a global basis, however, increasing agricultural intensification combined with an increased demand for food, feed and biofuel crops has resulted in shorter and less diverse crop rotations. Our analysis revealed the inclusion of legumes in the crop rotations to impact differences in microbial community abundance via an enhancement of microbial biomass nitrogen (Fig 4B). Furthermore, we identified enhanced microbial nitrogen in organic systems, exclusively when the two systems were under different rotations (Fig 4B). This indicates that different crops in the rotation, with a possible inclusion of legumes, increase microbial biomass nitrogen.

When comparing systems under similar rotations, the results showed urease activity to be higher under organic management. In contrast, no differences in urease activity were observed in systems under dissimilar rotations (Fig 4F). This indicates plant species effects to be potentially stronger than farming system effects. Our findings correspond with a recent meta-analysis by McDaniel et al. (2014), who synthesized crop rotation effects on soil carbon and nitrogen pools from 122 studies [67]. They identified the addition of one or more crops in the rotation to be beneficial in regard to microbial biomass carbon, microbial biomass nitrogen, total carbon and total nitrogen. Additionally, they showed the inclusion of cover crops enhances total nitrogen and carbon. Venter and his colleagues (2016) recently conducted a meta-analysis to assess the impact of aboveground biodiversity on microbial richness and diversity using molecular techniques and biochemical fingerprinting [68]. They show a diverse crop rotation to have a positive effect on microbial richness and diversity—though inclusion of legumes did not specifically affect microbial community structure.

Pesticides are a core component of modern agriculture used to suppress weeds, pests and diseases. Monocultures or very short rotations, in particular, lead to a high pest pressure [69], making the use of pesticides inevitable to secure yields. A wide range of different products exist, of which the majority are synthetically produced; although there are some which are based on natural products [70]. Both conventional and to a lesser extent organic farming depend on pesticides, though the systems are subjected to different regulations. Organic farming exclusively allows the use of pesticides which are of natural origin, whereas synthetically produced products may be applied in conventional farming systems. A review by Bünemann et al. (2006) synthesised the varying effects of several pesticides on microbial communities and other soil organisms [71]. However, organic pesticides are still widely considered to be less harmful to the environment than synthetic products, though several studies dispute this [72,73]. In our analysis, differences in microbial activity and community abundance were not impacted by different kind of pesticides (S4 Table). This indicates differences in pesticide effects to be rather weak or pesticide-effects themselves to be short. We therefore surmise that sampling date might have a crucial influence on the results. For future research in this regard, one should consider a subdivision of pesticides into herbicides, insecticides and fungicides combined with community structure data.

In addition, land use and plant life cycle were related to microbial activity and abundance. One interesting result was the effect of land use on the metabolic quotient, which did not differentiate well between the farming systems in overall meta-analysis, but did differentiate when sugrouped in orchards and grasslands. (Figs 2 and 4). The metabolic quotient serves as an indicator for microbial stress or disturbance as microorganisms divert more energy from growth into maintenance [17]. Our categorical analysis revealed orchards and more broadly also perennial organic systems to have a lower metabolic quotient compared to conventional systems. One explanation might be a higher disturbance and stress background in arable and annual systems due to tillage and fertilization, which subsequently undermines farming system effects in regard to the metabolic quotient. Therefore, the metabolic quotient might not serve as a suitable key indicator in order to differentiate organic and conventional arable farming.

Unfortunately, the effect of tillage practices on differences in microbial abundance and activity of organic and conventional farming systems could not be analysed due to a lack of primary studies. Zuber and Vilamils (2016) identified different tillage methods to affect microbial community abundance and activity in a global meta-analysis [74]. Whether these effects are enhanced or diminished in organic systems compared to conventional farming needs to be addressed in future primary research such as Sun et al. (2016) recently did in the well-known long-term trial Scheyern in Germany [75]. In addition, Larsen and co-workers [76] identified a positive effect of no-till in organic and conventional systems in regard to total carbon and microbial biomass carbon. The effect was higher in the organic systems but accompanied with lower yields probably due to enhanced weed pressure. Especially in cases where no-till is applied in organic farming further research regarding weed management and regulation is needed to improve yields [77].

In summary, key indicators for microbial community abundance and activity are not only enhanced in organic systems compared to conventional ones on an overall basis, but are also affected by different factors such as farming practices, land use and climatic conditions. In some contexts, the beneficial effect of organic farming on microbial abundance and activity vanishes and the two systems are comparable in certain target variables, whereas another target variable does not differentiate between the systems until certain categories are applied. Interestingly, differences in key indicators for microbial abundance and activity are impacted unequally by different contexts.

#### 3.3 Farming system induced properties affect microbial activity and abundance

To detect farming system induced properties potentially influencing microbial abundance and activity, and in order to validate our dataset a meta-analysis comparing SOC, TN and pH of all included comparisons was performed. The results were not meant to identify new findings, but rather to validate and compare our dataset with other previously published results like the meta-analysis performed by Gattinger et al. (2012) concerning carbon stocks in organic farming [39]. In the current study, farming system induced SOC was elevated by 19% on average in organic systems when compared to conventional systems (Table 2), which is mainly due to organic inputs as found by Gattinger and colleagues. In our study, meta-regression identified SOC to have a positive association with microbial biomass as discussed previously (S5 Table). An overall pH decrease of 2%, resulting in approximately 0.15 pH units, in conventional systems compared to organic systems was identified (Table 2). This acidification is most likely due to the application of synthetic fertilizers in conventional systems and was already described in several long-term fertilizer and farming system trials [26,78,79]. A study by Baath et al. (2003) identified a strong positive correlation between microbial biomass and pH in forest soils [80]. Pietri and colleagues (2008) [81] used a unique natural soil pH gradient at an experimental field, the Hoosfield acid strip, to analyse how microbial properties depend on pH [81]. The metabolic quotient at both ends of the pH scale showed the highest values, indicating stressful conditions for microorganisms. Furthermore, they described microbial biomass to be highest at around 5.7 pH and basal respiration to increase as pH values rise. In our study, the meta-regression displayed a higher pH of organic systems to positively influence microbial activity, whereas no relation to size could be identified.

In short, we found evidence for a positive influence of organic management on soil pH, which combined with enhanced SOC due to organic fertilisation, might create a more beneficial environment for microbial communities compared to more acidified conventional soils with lower SOC.

It has to be stated that no beneficial effect of conventional farming compared to organic was identified in any target variable, neither in overall meta-analysis, in contextual categorical meta-analyses nor in meta-regression when compared to organic.

Lastly, it should be noted that meta-analysis is an observational tool which highly depends on the quality and selection of primary studies. All exploratory attempts as done using categorical meta-analyses and meta-regressions have to be handled cautiously and are more an indicative measure than a proof of concept.

### 4. The effect of farming systems on microbial community structure

Apart from soil microbial community abundance and activity, also microbial community structure is important when thinking of sustainable provisioning of soil-based ecosystem services. A large and active microbial community which is low in functional diversity might not manage to adapt to new climatic conditions, whereas a diverse community could provide better resilience to environmental changes. In our study, the aspect of microbial community structure was not followed in a quantitative manner for several reasons. First, the amount of data associated with comparative analyses of community structures of organic and conventionally farmed systems suffice for a complete study on its own. Second, quantifying community structure differences is a challenging approach since the techniques from the 1990’s to the 2010s have changed drastically in their methodology and resolution, making a fully quantitative approach including all available studies a challenging undertaking.

However, systematic literature search of studies comparing microbial community structure of organic and conventional systems yielded 21 studies from all over the world, including long-term trials and farm comparison. The methods covered in these studies ranged from denaturing gradient gel electrophoresis (DGGE), terminal restriction fragment lengths polymorphism (TRFLP), phospholipid fatty-acids (PLFA), to GeoChip and next generation sequencing (NGS). The vast majority of the studies described a difference in community structure to occur [24,25,27,29,82–95], whereas a minority did not [96–98] (data not shown). To determine whether or not these differences favoured the organic or the conventional systems in terms of species diversity and richness, and which farming practices or climatic conditions have a positive or negative influence still requires future analysis of the individual studies.

### 5. Consequences for agricultural sustainability

Conventionally farmed agricultural systems strongly depend on potentially environmental harmful and highly energy consuming processes which is not only problematic in regard to global climate change but also considering peak oil. Further negative aspects of conventional agriculture are enhaced losses in biodiversity and soil degradation [5], the ongoing depletion of SOC [99,100] and the emission of potent greenhouse gases such as nitrous oxide [101].

In order to stop accelerating agricultural-mediated global climate change and its consequences, negative impacts of agricultural systems have to be reduced and replaced by alternatives. One option is reducing or eliminating the use of synthetic nitrogen fertilizers. Instead, the application of organic fertilizers in form of manures, slurries or other organic residues and the cultivation of leguminous crops should be encouraged. Aside from reducing energy demanding processes and environmental harmful effects such as nitrogen and phosphorus leaching, the use of organic fertilizers also enriches soils with SOC and thus might ameliorate SOC depletion [39]. In this regard, organic farming might represent a possible answer for eco-friendly agricultural intensification in order to meet the growing demand for food, fodder and biofuels while simultaneously coping with challenging climatic conditions and reducing the exploitation of Earth’s limited resources and other environmental impacts.

The present study identified a positive impact of organic farming and the underlying management practices on most in this study investigated microbial indicators which are likely linked to key soil functions such as nutrient cycling andsoil structure formation.

Nevertheless, organic farming nowadays still produces on average 20% less yield compared to conventional (data mainly originating from the Northern hemisphere) [41,54], mainly due to nitrogen shortage, diseases, pests and the cultivation of non-adapted crop varieties. However, there is evidence for organic systems to outperform conventional under more extreme weather scenarios [56] and thus considering future climate changes, the yield gap might shrink as well.

The application of selected farming practices such as the incorporation of perennials, legumes, the extension of crop rotations, or the application of organic fertilizers, should also be considered in conventional systems to promote microbial abundance and activity and thus to enhance ecosystem services such as nutrient cycling, soil structure formation and regulation of pests and diseases.

Besides organic farming, there are other management strategies, which can positively affect the investigated effect sizes and associated soil functions. Long-term reduced tillage and no-tillage practices have been identified to positively act on microbial indicators such as fungal hyphae length, and fungal abundance [102] but also on soil aggregate structure [103] and soil chemical properties [104]. The number of farms performing reduced and no-tillage practices, especially in Europe, is rather low (http://ec.europa.eu/eurostat/), as it is for organic farming, and thus the potential to grow exist for both practices [105]. The combination of organic farming with reduced or no-tillage is challenging, but has been shown to work as well [77,106]. Nevertheless, both options might improve biological but also physical and chemical soil parameters, which are highly relevant considering climate change induced rainfall variabilities and soils’ enhanced need for water infiltration and storage.

To deepen the understanding of certain impact factors like climatic conditions or tillage on the observed differences in microbial traits between organic and conventional systems, more investigations are needed to drive a more complete picture, especially in the context of global climate change.

Overall, it can be concluded that organic management practices positively impact most of the investigated microbial indicators. Whether or not these observed differences as well translate into an extended capacity to adapt to climate change is still rarely examined and currently investigated in the European joint projects ‘Sustainable provisioning of multiple ecosystem services in agricultural landscapes’ (ECO-SERVE) and ‘Managing soil biodiversity and ecosystem services in agroecosystems across Europe under climate change’ (SOILCLIM).

## Supporting information

S1 Fig PRISMA flowdiagramm. Overview of identified, excluded and included studies.
(TIF)

S1 Table Overview of all studies used in the meta-analysis, their characteristics and target variables contributed. Sites including multiple farming system comparisons might appear in different studies several times independently investigating different target variables.
(DOCX)

S2 Table Results of the overall meta-analysis for the seven effect sizes. Random effects model with a 95% confidence interval (CI) and a Z-distribution were applied. I^2^ estimates the amount of heterogeneity in the dataset based on true between study variance from 0% to a 100%.
(DOCX)

S3 Table Overview of Duvall and Tweedie’s trim and fill sensitivity analysis. Observed values are from meta-analysis with the original dataset whereas adjusted values are from corrected datasets according to Duvall and Tweedie’s trimm and fill. RR = response ratio; n = sample size.
(DOCX)

S4 Table Results of the categorical meta-analysis. Categorical random effects model with a 95% confidence interval (CI) was applied. n.s. = non-significant, n = sample size. Köppen climatic zones are abbreviated as followed: A = Tropical/megathermal climates, B = Dry climates, C = Temperate/Mesothermal climates, D = Continental/Microthermal climates). No = ‘no legumes’/‘no organic input’ in both farming systems. ORG = organic, CON = conventional/non-organic systems.
(DOCX)

S5 Table Results of the meta-regression based on farming system induced factors. Random effects model with Knapp-Hartung adjustments were applied with the listed covariates. All computational procedures were done using logarithmized data to obtain a normally distributed dataset.
(DOCX)

S6 Table PRISMA checklist. (DOCX)

S7 Table Minimal data set. (DOCX)
