# Supplementary figures and images for: Organic farming enhances soil microbial abundance and activity—A meta-analysis and meta-regression
Source: PLoS One. 2017 Jul 12;12(7):e0180442. doi: 10.1371/journal.pone.0180442 (PMC5507504; doi:10.1371/journal.pone.0180442)

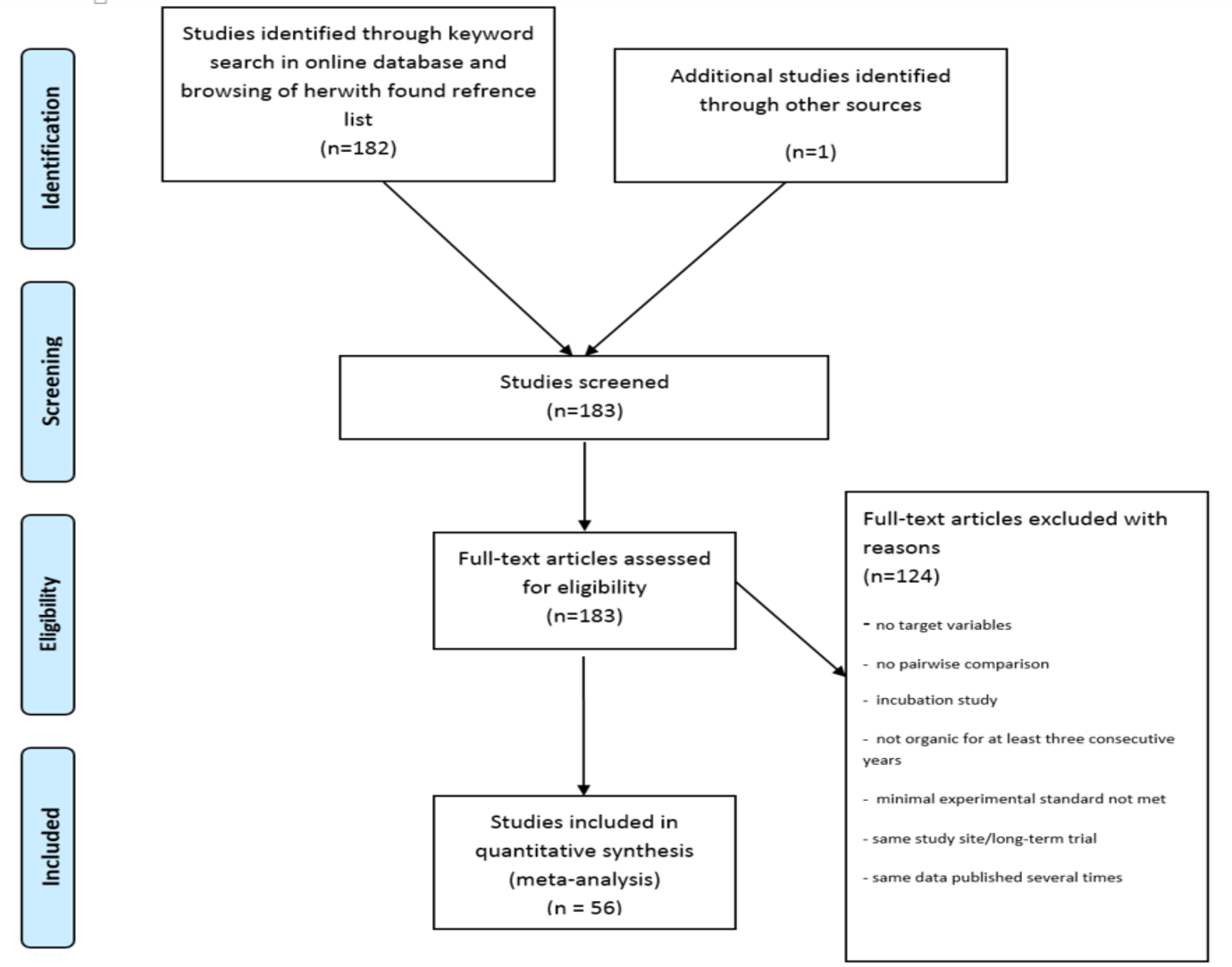

Supplement: S1 Fig — Overview of identified, excluded and included studies. (TIF) [file pone.0180442.s001.tif]
