# Supplementary material for: Organic farming enhances soil microbial abundance and activity—A meta-analysis and meta-regression
Source: PLoS One. 2017 Jul 12;12(7):e0180442. doi: 10.1371/journal.pone.0180442 (PMC5507504; doi:10.1371/journal.pone.0180442)
Supplement: S1 Table — Sites including multiple farming system comparisons might appear in different studies several times independently investigating different target variables. (DOCX) [file pone.0180442.s002.docx]

| **Author/Study** | **Location (Site and/or Town, Country)** | **# of comparisons** | **Set up** | **Target variables included** |  |  |
| --- | --- | --- | --- | --- | --- | --- |
| Andrews *et al.,*2002 | Between Mendota and Huron (CA), USA | 1 | Farm comparison | Microbial C, Microbial N |  |  |
| Anonymous, unpublished | Chukha and Tika, Kenya | 2 | Experimental plots | Microbial C, Microbial N, Metabolic quotient, Dehydrogenase activity | | |
| Aparna *et al*., 2014 | Village Dhaban, Taluka Sangaria, Rajasthan, India | 2 | Farm comparison | Dehydrogenase activity |  |  |
| Bending *et al.,* 2004 | HRI-Wellesbourne farm, Warwickshire, UK | 4 | Experimental plots | Basalrespiration |  |  |
| Benitez *et al.*, 2006 | Southern Spain, Spain | 2 | Farm comparison | Dehydrogenase activity |  |  |
| Bolton Jr *et al.*, 1985 | Spokane, Washington, USA | 1 | Farm comparison | Microbial C, Dehydrogenase activity, Urease activity | |  |
| Bossio *et al*., 1998 | Long-term Research on Agricultural Systems (LTRAS), Davis (CA), USA | 2 | Experimental plots | Microbial C, Microbial N, Basalrespiration, Metabolic quotient | | |
| Burger *et al.,* 2005 | Long-term Research on Agricultural Systems (LTRAS), Davis (CA), USA | 1 | Experimental plots | Microbial C, total PLFA, Basalrespiration, Metabolic quotient | | |
| Carey *et al.,* 2009 | Te Puke area, New Zealand | 2 | Farm comparison | Microbial C, Microbial N, Basalrespiration, Metabolic quotient | | |
| Carpenter-Boggs *et al.*, 2000 | Palouse Concervation Farm and Spillman Research Farm, Washington Sate University, USA | 2 | Experimental plots | Microbial C, Basalrespiration, Metabolic quotient, Dehydrogenase activity | | |
| Chirinda *et al.*, 2010 | Fouloum, Denmark | 8 | Experimental plots | Microbial N, Basalrespiration |  |  |
| Esperschütz *et al*., 2007 | DOK, Therwil, Switzerland | 4 | Experimental plots | Total PLFA |  |  |
| Fliessbach *et al*., 2007 | DOK, Therwil, Switzerland | 4 | Experimental plots | Basalrespiration, Dehydrogenase activity | |  |
| Forlan-Amaral *et al*., 2012 | Parana, Brazil | 4 | Experimental plots | Microbial C, Basalrespiration, Metabolic quotient | |  |
| Fraser *et al*., 1989 | Agricultural Research and Developement Center at Mead, Nebraska | 1 | Experimental plots | Microbial C, Basalrespiration, Metabolic quotient | |  |
| Freitas *et al.,* 2011 | Petrolina, Brazil | 1 | Farm comparison | Microbial C, Basalrespiration, Metabolic quotient | |  |
| Gajda and Martyniuk, 2004 | Osiny Experimental Farm (ISSPC), Poland | 1 | Experimental plots | Micobial C, Basalrespiration, Metabolic quotient, Dehyydrogenase activity | | |
| Garcia-Ruiz et al 2009 | Jaén, Southern Spain | 2 | Farm comparison | Dehydrogenase activity |  |  |
| Ge et al. 2011 | Beigang Horticulture Farms, Shanghai, China | 2 | Experimental plots | Microbial C, Microbial N, Basalrespiration, Metabolic quotient, Urease avctivity | | |
| Ge et al. 2013 | Jichang Town, Pudong district, China | 2 | Experimental plots | Microbial C, Microbial N, total PLFA |  |  |
| Glover et al. 2000 | Yakima Valley of Washington state, USA | 2 | Experimental plots | Microbial C, Microbial N |  |  |
| Gonzales-Perez et al. 2015 | Chonbentang, Shanghai, China | 1 | Experimental plots | Microbial C, Microbial N |  |  |
| Gunapala et al. 1998 | Agronomy Field Facility, Davis, USA | 1 | Experimental plots | Microbial C, Microbial N |  |  |
| Hartmann et al. 2006 | DOK, Therwil, Switzerland | 4 | Experimental plots | Microbial C |  |  |
| Heinze et al. 2010 | Institute of Biodynamic Research, Darmstadt, Germany | 2 | Experimental plots | Microbial C, Microbial N, Basalrespiration, Metabolic quotient | | |
| Islam and Weil 2000 | Rodale Institute Reearch Center, Pennsylvania, USA | 2 | Experimental plots | Microbial C, Basalrespiration, Metabolic quotient | |  |
| Jacinthe et al. 2011 | Anthony Village, New Mexico | 3 | Farm comparison | Microbial C, Basalrespiration, Metabolic quotient | |  |
| Järvan et al. 2014 | Olustvere, Estonia | 2 | Experimental plots | Dehydrogenase activity |  |  |
| Kong et al., 2011 | Long-term Research on Agricultural Systems (LTRAS), Russel Ranch, Davis, CA, USA | 2 | Experimental plots | Total PLFA |  |  |
| Lagomarsino et al. 2009 | University of Tuscia experimental farm, Viterbo, Italy | 3 | Experimental plots | Microbial C, Basalrespiration, Metabolic quotient, Dehydrogenase activity | | |
| Larsen et al. 2014 | Mountain Horticulture Crops Research Station, North Carolina, USA | 4 | Experimental plots | Microbial C, Microbial N |  |  |
| Liebig and Doran 1999 | Nebraska, USA | 5 | Farm comparison | Microbial C, Microbial N |  |  |
| Marinari et al. 2006 | ColleValle Agrinatura farm, Italy | 1 | Farm comparison | Microbial C, Microbial N, Dehydrogenase activity, Protease activity | | |
| Marinari et al. 2010 | Colle Valle, Viterbo, field comparison; DOK, Therwil, Switzerland; La Selva, Italy | 3 | Farm comparison/Experimental plots (different sites) | Basalrespiration |  |  |
| Marinari et al. 2010 B | University of Tuscia experimental farm, Viterbo, Italy | 1 | Experimental plots | Microbial N, Microbial C, Basalrespiration, Metabolic quotient | | |
| Mazzoncini et al. 2010 | MASCOT, CIRAA, Universuty of Pisa, Italy | 1 | Experimental plots | Microbial C, Basalrepsiration, Metabolic quotient | |  |
| Melero et al. 2006 | Las Torres-Tomejil farm, Sevilla, Spain | 3 | Experimental plots | Microbial C, Microbial N, Basalrepsiration, Metabolic quotient, Urease activity | | |
| Migliorini et al. 2014 | MOLTE (Montepaldi Longterm Experiment), University of Florence, Italy | 2 | Experimental plots | Microbial C, Metabolic quotient |  |  |
| Moeskops et al. 2010 | Java, Indonesia | 6 | Farm comparison | Microbial C, total PLFA, Dehydrogenase activity | |  |
| Monokrousos et al. 2006 | Kria Vrisi, Greece | 3 | Farm comparison | Microbial C, Microbial N, Basalrespiration, Metabolic quotient, Urease activity | | |
| Murata et al. 1997 | Rakaia, Canterburry, New Zealand | 2 | Farm comparison | Microbial C, Microbial N |  |  |
| Nguyen et al. 1995 | Temuka, New Zealand | 6 | Farm comparison | Urease activity |  |  |
| Oberholzer et al. 2000 | Farm comparison, Switzreland | 1 | Farm comparison | Microbial C, Metabolic quotient |  |  |
| Okur et al. 2009 | Manisa, Turkey | 3 | Farm comparison | Microbial C, Protease activty, Urease activity, Dehydrogenase activty | | |
| Padmavathy and Poyyamoli 2011 | Puducherry, India | 1 | Farm comparison | Microbial C |  |  |
| Petersen et al. 1997 | Denmark | 8 | Farm comparison | Microbial C |  |  |
| Reganold et al. 1993 | North Island of New Zealand | 7 | Farm comparison | Basalrespiration |  |  |
| Reganold et al. 2010 | Watsonville area, California, USA | 1 | Farm comparison | Microbial C, Basalrespiration, Metabolic quotient, Protease activity, Dehydrogenase activity | | |
| Romaniuk et al. 2011 | La Plata, Buenos Aires, Argentinia | 2 | Farm comparison | Total PLFA |  |  |
| Schjønning et al. 2002 | Fouloum, Denmark | 4 | Farm comparison | Microbial C |  |  |
| Stark et al. 2008 | Lincoln University New Zealand | 2 | Experimental plots | Microbial C, Dehydrogenase activity |  |  |
| Sudhakaran et al 2013 | Pondicherry, Coramandel coast, India | 2 | Farm comparison | Basalrespiration |  |  |
| Van Diepeningen et al. 2006 | Netherlands | 2 | Farm comparison | Basalrespiration |  |  |
| Velmourougane 2016 | Chettalli, Karnataka, India | 1 | Experimental plots | Basalrespiration, Urease activity, Dehydrogenase activity | | |
| Wander et al. 1995 | Rodale Institute Reearch Center, Pennsylvania | 2 | Experimental plots | total PLFA, Basalrespiration |  |  |
| Wells et al. 2000 | Somersby, Australia | 3 | Experimental plots | Microbial C |  |  |
| Yeates et al. 1997 | Glanrhyd in Dyfeld, Wales | 3 | Farm comparison | Microbial C, total PLFA, Dehydrogenase activity | |  |
