# Supplementary material for: Organic farming enhances soil microbial abundance and activity—A meta-analysis and meta-regression
Source: PLoS One. 2017 Jul 12;12(7):e0180442. doi: 10.1371/journal.pone.0180442 (PMC5507504; doi:10.1371/journal.pone.0180442)
Supplement: S2 Table — Random effects model with a 95% confidence interval (CI) and a Z-distribution were applied. I2 estimates the amount of heterogeneity in the dataset based on true between study variance from 0% to a 100%. (DOCX) [file pone.0180442.s003.docx]

|  | **RR** | **Lower 95% CI** | **Upper 95% CI** |  | **Z-value** | **2-sided p-value** | **Tau²** | **Tau** | **I²** | **Q** | **df** | **n** | **p** |
| --- | --- | --- | --- | --- | --- | --- | --- | --- | --- | --- | --- | --- | --- |
| Microbial biomass carbon | 1.41 | 1.30 | 1.52 |  | 8.61 | 0.000 | 0.10 | 0.32 | 82 | 545.62 | 99 | 100 | ≤0.001 |
| Microbial biomass nitrogen | 1.51 | 1.29 | 1.76 |  | 5.21 | 0.000 | 0.26 | 0.51 | 96 | 1195.84 | 48 | 49 | ≤0.001 |
| Total PLFA | 1.59 | 1.39 | 1.81 |  | 6.94 | 0.000 | 0.08 | 0.28 | 91 | 233.91 | 21 | 22 | ≤0.001 |
| Dehydrogenase activity | 1.74 | 1.52 | 1.98 |  | 8.17 | 0.000 | 0.10 | 0.32 | 72 | 141.52 | 39 | 40 | ≤0.001 |
| Protease activity | 1.84 | 1.63 | 1.98 |  | 9.81 | 0.000 | 0.01 | 0.12 | 54 | 13.06 | 6 | 7 | ≤0.001 |
| Urease activity | 1.32 | 1.16 | 1.50 |  | 4.24 | 0.000 | 0.06 | 0.25 | 90 | 178.20 | 17 | 18 | ≤0.001 |
| Metabolic quotient | 0.96 | 0.84 | 1.09 |  | -0.63 | 0.529 | 0.14 | 0.37 | 89 | 340.34 | 39 | 40 | 0.142 |
