# Supplementary material for: Organic farming enhances soil microbial abundance and activity—A meta-analysis and meta-regression
Source: PLoS One. 2017 Jul 12;12(7):e0180442. doi: 10.1371/journal.pone.0180442 (PMC5507504; doi:10.1371/journal.pone.0180442)
Supplement: S3 Table — Observed values are from meta-analysis with the original dataset whereas adjusted values are from corrected datasets according to Duvall and Tweedie’s trimm and fill. RR = response ratio; n = sample size. (DOCX) [file pone.0180442.s004.docx]

|  |  |  | **studies adjusted** | |  |
| --- | --- | --- | --- | --- | --- |
| **Microbial biomass carbon** | | **RR** | **to left of mean** | **to right of mean** | **n** |
|  | observed values | 1.40 |  |  | 100 |
|  | adjusted values | 1.51 | 0 | 11 | 111 |
| **Microbial biomass nitrogen** | | |  |  |  |
|  | observed values | 1.51 |  |  | 49 |
|  | adjusted values | 1.90 | 12 | 0 | 61 |
| **Total PLFA** | |  |  |  |  |
|  | observed values | 1.59 |  |  | 22 |
|  | adjusted values | 1.75 | 0 | 5 | 27 |
| **Basalrespiration** | |  |  |  |  |
|  | observed values | 1.20 |  |  | 66 |
|  | adjusted values | 1.05 | 17 | 0 | 83 |
| **Dehydrogenase activity** | |  |  |  |  |
|  | observed values | 1.74 |  |  | 40 |
|  | adjusted values | 1.74 | 0 | 0 | 40 |
| **Metabolic quotient** | |  |  |  |  |
|  | observed values | 0.984 |  |  | 40 |
|  | adjusted values | 0.987 | 0 | 2 | 42 |
| **Protease activity** | |  |  |  |  |
|  | observed values | 1.84 |  |  | 7 |
|  | adjusted values | 1.84 | 0 | 0 | 7 |
| **Urease activity** | |  |  |  |  |
|  | observed values | 1.32 |  |  | 18 |
|  | adjusted values | 1.32 | 0 | 0 | 18 |
