# Supplementary material for: Organic farming enhances soil microbial abundance and activity—A meta-analysis and meta-regression
Source: PLoS One. 2017 Jul 12;12(7):e0180442. doi: 10.1371/journal.pone.0180442 (PMC5507504; doi:10.1371/journal.pone.0180442)
Supplement: S4 Table — Categorical random effects model with a 95% confidence interval (CI) was applied. n.s. = non-significant, n = sample size. Köppen climatic zones are abbreviated as followed: A = Tropical/megathermal climates, B = Dry climates, C = Temperate/Mesothermal climates, D = Continental/Microthermal climates). No = ‘no legumes’/‘no organic input’ in both farming systems. ORG = organic, CON = conventional/non-organic systems. (DOCX) [file pone.0180442.s005.docx]

|  |  | **Microbial biomass carbon** | | | | | |
| --- | --- | --- | --- | --- | --- | --- | --- |
|  |  | **RR** | **CI Lower** | **CI Upper** | **Z-value** | **n** | **p-value** |
| **Full dataset** |  | 1.58 | 1.52 | 1.65 | 21.54 | 100 | ≤0.001 |
| **Climate zone (Köppen)** | **A** | 1.70 | 1.59 | 1.83 | 14.67 | 8 | ≤0.001 |
|  | **B** | 1.42 | 1.19 | 1.70 | 3.95 | 11 | ≤0.001 |
|  | **C** | 1.43 | 1.28 | 1.60 | 6.41 | 63 | ≤0.001 |
|  | **D** | 1.29 | 1.10 | 1.52 | 3.06 | 10 | 0. |
| **Continent** | **Africa** | 1.12 | 0.69 | 1.81 | 0.44 | 2 | no data |
|  | **Asia** | 1.91 | 1.53 | 2.39 | 5.69 | 15 | ≤0.001 |
|  | **Europe** | 1.28 | 1.17 | 1.41 | 5.19 | 41 | ≤0.001 |
|  | **North America** | 1.37 | 1.20 | 1.56 | 4.76 | 27 | ≤0.001 |
|  | **Oceania** | 1.16 | 0.93 | 1.45 | 1.33 | 10 | n.s. |
|  | **South America** | 2.13 | 1.54 | 2.95 | 4.58 | 5 | ≤0.001 |
| **Landuse** | **Arable** | 1.40 | 1.30 | 1.51 | 8.84 | 84 | ≤0.001 |
|  | **Grassland** | 0.91 | 0.57 | 1.47 | -0.38 | 4 | n.s. |
|  | **Orchard** | 1.63 | 1.30 | 2.04 | 4.28 | 12 | ≤0.001 |
| **Plant life cycle** | **Annual** | 1.40 | 1.30 | 1.51 | 8.93 | 85 | ≤0.001 |
|  | **Perennial** | 1.47 | 1.08 | 1.99 | 2.48 | 15 | 0.001 |
| **Set up** | **Field/Farm** | 1.54 | 1.38 | 1.71 | 7.84 | 57 | ≤0.001 |
|  | **Longterm** | 1.21 | 1.12 | 1.31 | 4.75 | 3 | ≤0.001 |
| **Time since conversion** | **4-10 years** | 1.36 | 1.18 | 1.57 | 4.17 | 36 | ≤0.001 |
|  | **<10 years** | 1.35 | 1.25 | 1.47 | 7.32 | 37 | ≤0.001 |
|  | **3 years** | 1.53 | 1.09 | 2.14 | 2.47 | 5 | 0.013 |
| **Clay content** | **0-15%** | 1.41 | 1.29 | 1.54 | 7.81 | 27 | ≤0.001 |
|  | **16-40%** | 1.67 | 1.36 | 2.04 | 4.98 | 27 | ≤0.001 |
|  | **41-100%** |  |  |  |  | 0 | no data |
| **Croprotation** | **Different** | 1.53 | 1.32 | 1.78 | 5.56 | 44 | ≤0.001 |
|  | **Similar** | 1.34 | 1.20 | 1.51 | 5.01 | 37 | ≤0.001 |
| **Legumes in Croprotation in:** | **no** | 1.57 | 1.30 | 1.89 | 4.65 | 23 | ≤0.001 |
|  | **ORG + CON** | 1.29 | 1.16 | 1.44 | 4.55 | 44 | ≤0.001 |
|  | **ORG** | 1.56 | 1.24 | 1.97 | 4.55 | 24 | ≤0.001 |
|  | **CON** | 2.25 | 1.43 | 3.56 | 3.48 | 1 | no data |
| **Oranic Inputs in:** | **no** | 0.95 | 0.68 | 1.31 | -0.33 | 5 | n.s. |
|  | **ORG + CON** | 1.21 | 1.06 | 1.37 | 2.84 | 25 | 0.005 |
|  | **ORG** | 1.57 | 1.43 | 1.72 | 9.65 | 63 | ≤0.001 |
|  | **CON** | 1.30 | 0.99 | 1.71 | 1.89 | 1 | no data |
| **Use of synthetic pesticides** | **CON** | 1.49 | 1.33 | 1.66 | 7.11 | 55 | ≤0.001 |
|  | **no** | 2.19 | 1.29 | 3.73 | 2.90 | 4 | ≤0.001 |
|  |  |  |  |  |  |  |  |
|  |  | **Microbial biomass nitrogen** | | | | | |
|  |  | **Point** | **CI Lower** | **CI Upper** | **Z-value** | **n** | **p-value** |
| **Full dataset** |  | 1.35 | 1.24 | 1.47 | 6.76 | 49 | ≤0.001 |
| **Climate zone (Köppen)** | **A** | 1.26 | 0.91 | 1.73 | 1.40 | 1 | no data |
|  | **B** | 1.35 | 1.05 | 1.74 | 2.32 | 4 | 0.020 |
|  | **C** | 1.62 | 1.29 | 2.03 | 4.15 | 29 | ≤0.001 |
|  | **D** | 1.30 | 1.17 | 1.45 | 4.83 | 15 | ≤0.001 |
| **Continent** | **Africa** | 1.01 | 0.67 | 1.54 | 0.07 | 2 | no data |
|  | **Asia** | 3.00 | 1.78 | 5.08 | 4.10 | 5 | ≤0.001 |
|  | **Europe** | 1.15 | 0.99 | 1.34 | 1.83 | 18 | n.s. |
|  | **North America** | 1.68 | 1.37 | 2.05 | 5.02 | 20 | ≤0.001 |
|  | **Oceania** | 1.32 | 1.20 | 1.45 | 5.74 | 4 | ≤0.001 |
|  | **South America** |  |  |  |  | 0 |  |
| **Landuse** | **Arable** | 1.53 | 1.29 | 1.81 | 4.85 | 43 | ≤0.001 |
|  | **Grassland** | 1.13 | 0.78 | 1.63 | 0.66 | 1 | no data |
|  | **Orchard** | 1.42 | 1.13 | 1.79 | 3.00 | 5 | 0.005 |
| **Plant life cycle** | **Annual** | 1.53 | 1.30 | 1.82 | 4.97 | 44 | ≤0.001 |
|  | **Perennial** | 1.30 | 1.06 | 1.59 | 2.49 | 5 | * |
| **Set up** | **Field/Farm** | 1.61 | 1.34 | 1.94 | 5.04 | 26 | ≤0.001 |
|  | **Longterm** | 1.30 | 1.06 | 1.60 | 2.56 | 22 | 0.003 |
| **Time since conversion** | **4-10 years** | 1.34 | 1.20 | 1.50 | 5.17 | 34 | ≤0.001 |
|  | **<10 years** | 1.89 | 1.38 | 2.57 | 3.99 | 12 | ≤0.001 |
|  | **3 years** | 0.70 | 0.57 | 0.85 | -3.65 | 1 | no data |
| **Clay content** | **0-15%** | 1.35 | 1.09 | 1.68 | 2.70 | 4 | 0.004 |
|  | **16-40%** | 1.75 | 1.16 | 2.65 | 2.66 | 11 | 0.004 |
|  | **41-100%** |  |  |  |  | 0 |  |
| **Croprotation** | **Different** | 1.74 | 1.37 | 2.20 | 4.61 | 22 | ≤0.001 |
|  | **Similar** | 1.17 | 0.99 | 1.37 | 1.86 | 17 | n.s. |
| **Legumes in Croprotation in:** | **no** | 1.26 | 0.93 | 1.71 | 1.48 | 10 | n.s. |
|  | **ORG + CON** | 1.29 | 1.18 | 1.41 | 5.43 | 22 | ≤0.001 |
|  | **ORG** | 2.01 | 1.46 | 2.75 | 4.31 | 16 | ≤0.001 |
|  | **CON** | 2.31 | 1.57 | 3.40 | 4.23 | 1 | no data |
| **Oranic Inputs in:** | **no** | 1.33 | 0.87 | 2.04 | 1.32 | 4 | n.s. |
|  | **ORG + CON** | 0.95 | 0.78 | 1.15 | -0.55 | 9 | n.s. |
|  | **ORG** | 1.71 | 1.43 | 2.05 | 5.81 | 30 | ≤0.001 |
|  | **CON** |  |  |  |  | 0 |  |
| **Use of synthetic pesticides** | **CON** | 1.61 | 1.25 | 2.08 | 3.70 | 28 | ≤0.001 |
|  | **no** | 1.87 | 1.44 | 2.45 | 4.62 | 3 | ≤0.001 |
|  |  |  |  |  |  |  |  |
|  |  | **Total PLFA** | | | | | |
|  |  | **Point** | **CI Lower** | **CI Upper** | **Z-value** | **n** | **p-value** |
| **Full dataset** |  | 1.73 | 1.57 | 1.91 | 11.14 | 22 | ≤0.001 |
| **Climate zone (Köppen)** | **A** | 1.83 | 1.64 | 2.04 | 10.78 | 6 | ≤0.001 |
|  | **B** |  |  |  |  | 0 |  |
|  | **C** | 1.49 | 1.19 | 1.86 | 3.51 | 14 | ≤0.001 |
|  | **D** | 1.16 | 0.66 | 2.05 | 0.51 | 2 | no data |
| **Continent** | **Africa** |  |  |  |  | 0 |  |
|  | **Asia** | 2.06 | 1.71 | 2.47 | 7.75 | 8 | ≤0.001 |
|  | **Europe** | 1.26 | 1.07 | 1.47 | 2.83 | 7 | 0.005 |
|  | **North America** | 1.34 | 1.07 | 1.66 | 2.58 | 4 | 0.010 |
|  | **Oceania** | 1.77 | 1.50 | 2.09 | 6.74 | 1 | no data |
|  | **South America** | 1.30 | 1.05 | 1.56 | 2.42 | 2 | no data |
| **Landuse** | **Arable** | 1.66 | 1.45 | 1.89 | 7.42 | 19 | ≤0.001 |
|  | **Grassland** | 1.24 | 0.86 | 1.81 | 1.14 | 3 | n.s. |
|  | **Orchard** |  |  |  |  | 0 |  |
| **Plant life cycle** | **Annual** | 1.66 | 1.45 | 1.89 | 7.42 | 19 | ≤0.001 |
|  | **Perennial** | 1.24 | 0.86 | 1.81 | 1.14 | 3 | n.s. |
| **Set up** | **Field/Farm** | 1.82 | 1.52 | 2.17 | 6.64 | 11 | ≤0.001 |
|  | **Longterm** | 1.37 | 1.24 | 1.52 | 6.00 | 11 | ≤0.001 |
| **Time since conversion** | **4-10 years** | 1.88 | 1.52 | 2.33 | 5.80 | 9 | ≤0.001 |
|  | **<10 years** | 1.41 | 1.26 | 1.59 | 5.82 | 8 | ≤0.001 |
|  | **3 years** | 1.85 | 1.65 | 2.07 | 10.66 | 2 | no data |
| **Clay content** | **0-15%** | 1.28 | 1.11 | 1.47 | 3.32 | 4 | ≤0.001 |
|  | **16-40%** | 1.74 | 1.21 | 2.50 | 3.00 | 5 | 0.003 |
|  | **41-100%** | 1.80 | 1.35 | 2.41 | 3.95 | 1 | no data |
| **Croprotation** | **Different** | 1.85 | 1.45 | 2.38 | 4.86 | 7 | ≤0.001 |
|  | **Similar** | 1.54 | 1.37 | 1.74 | 7.20 | 13 | ≤0.001 |
| **Legumes in Croprotation in:** | **no** | 2.06 | 1.71 | 2.47 | 7.75 | 8 | ≤0.001 |
|  | **ORG + CON** | 1.27 | 1.14 | 1.43 | 4.13 | 10 | ≤0.001 |
|  | **ORG** | 1.57 | 1.28 | 1.91 | 4.43 | 4 | ≤0.001 |
|  | **CON** |  |  |  |  | 0 |  |
| **Oranic Inputs in:** | **no** | 0.91 | 0.71 | 1.17 | -0,737 | 1 | no data |
|  | **ORG + CON** | 1.70 | 1.53 | 1.90 | 9.65 | 9 | ≤0.001 |
|  | **ORG** | 1.62 | 1.28 | 2.05 | 4.07 | 11 | ≤0.001 |
|  | **CON** | 1.20 | 0.93 | 1.54 | 1.30 | 1 | no data |
| **Use of synthetic pesticides** | **CON** | 1.68 | 1.45 | 1.95 | 6.81 | 16 | ≤0.001 |
|  | **no** |  |  |  |  | 0 |  |
|  |  |  |  |  |  |  |  |
|  |  | **Dehydrogenase activity** | | | | | |
|  |  | **Point** | **CI Lower** | **CI Upper** | **Z-value** | **n** | **p-value** |
| **Full dataset** |  | 1.66 | 1.483 | 1.85 | 8.95 | 40 | ≤0.001 |
| **Climate zone (Köppen)** | **A** | 3.15 | 1.69 | 5.89 | 3.60 | 8 | ≤0.001 |
|  | **B** | 2.09 | 1.66 | 2.63 | 6.26 | 10 | ≤0.001 |
|  | **C** | 1.58 | 1.37 | 1.83 | 6.21 | 18 | ≤0.001 |
|  | **D** | 1.22 | 0.92 | 1.63 | 1.40 | 4 | n.s. |
| **Continent** | **Africa** | 1.23 | 0.94 | 1.62 | 1.54 | 2 | no data |
|  | **Asia** | 2.40 | 1.83 | 3.14 | 6.36 | 12 | ≤0.001 |
|  | **Europe** | 1.51 | 1.27 | 1.80 | 4.62 | 20 | ≤0.001 |
|  | **North America** | 1.81 | 1.45 | 2.24 | 5.35 | 4 | ≤0.001 |
|  | **Oceania** | 1.51 | 0.79 | 2.88 | 1.26 | 2 | no data |
|  | **South America** |  |  |  |  | 0 |  |
| **Landuse** | **Arable** | 1.81 | 1.51 | 2.16 | 6.48 | 25 | ≤0.001 |
|  | **Grassland** | 1.22 | 0.84 | 1.77 | 1.02 | 3 | n.s. |
|  | **Orchard** | 1.85 | 1.50 | 2.29 | 5.73 | 12 | ≤0.001 |
| **Plant life cycle** | **Annual** | 1.81 | 1.51 | 2.16 | 6.48 | 25 | ≤0.001 |
|  | **Perennial** | 1.66 | 1.35 | 2.30 | 4.83 | 15 | ≤0.001 |
| **Set up** | **Field/Farm** | 1.81 | 1.52 | 2.17 | 6.56 | 26 | ≤0.001 |
|  | **Longterm** | 1.63 | 1.37 | 1.95 | 5.46 | 14 | ≤0.001 |
| **Time since conversion** | **4-10 years** | 1.71 | 1.48 | 1.98 | 7.30 | 23 | ≤0.001 |
|  | **<10 years** | 1.69 | 1.37 | 2.09 | 4.90 | 6 | ≤0.001 |
|  | **3 years** | 2.91 | 1.30 | 6.49 | 2.60 | 4 | 0.009** |
| **Clay content** | **0-15%** | 1.72 | 1.50 | 1.98 | 7.59 | 8 | ≤0.001 |
|  | **16-40%** | 1.54 | 1.12 | 2.12 | 2.65 | 12 | 0.008 |
|  | **41-100%** | 1.56 | 1.18 | 2.05 | 3.14 | 2 | no data |
| **Croprotation** | **Different** | 1.31 | 1.14 | 1.51 | 3.71 | 13 | ≤0.001 |
|  | **Similar** | 2.00 | 1.68 | 2.39 | 7.65 | 23 | ≤0.001 |
| **Legumes in Croprotation in:** | **no** | 2.20 | 1.70 | 2.85 | 5.95 | 13 | ≤0.001 |
|  | **ORG + CON** | 1.49 | 1.28 | 1.73 | 5.12 | 17 | ≤0.001 |
|  | **ORG** | 1.41 | 1.02 | 1.95 | 2.06 | 5 | 0.040 |
|  | **CON** |  |  |  |  | 0 |  |
| **Oranic Inputs in:** | **no** | 0.84 | 0.64 | 1.10 | -1.29 | 1 | no data |
|  | **ORG + CON** | 1.95 | 1.50 | 2.54 | 4.98 | 15 | ≤0.001 |
|  | **ORG** | 1.77 | 1.53 | 2.05 | 7.56 | 23 | ≤0.001 |
|  | **CON** | 1.40 | 1.06 | 1.85 | 2.37 | 1 | no data |
| **Use of synthetic pesticides** | **CON** | 1.38 | 1.57 | 2.14 | 7.66 | 31 | ≤0.001 |
|  | **no** | 2.12 | 1.29 | 3.48 | 2.98 | 1 | no data |
|  |  |  |  |  |  |  |  |
|  |  | **Metabolic quotient** | | | | | |
|  |  | **Point** | **CI Lower** | **CI Upper** | **Z-value** | **n** | **p-value** |
| **Full dataset** |  | 0.90 | 0.79 | 1.04 | -1,467 | 40 | n.s. |
| **Climate zone (Köppen)** | **A** | 0.69 | 0.53 | 0.90 | -2.73 | 1 | no data |
|  | **B** | 0.71 | 0.38 | 1.32 | -1.09 | 6 | 0.267 |
|  | **C** | 0.96 | 0.83 | 1.12 | 0.80 | 28 | 0.147 |
|  | **D** | 1.09 | 0.89 | 1.34 | 0.80 | 5 | 0.424 |
| **Continent** | **Africa** | 0.92 | 0.53 | 1.63 | -0.27 | 2 | no data |
|  | **Asia** | 3.69 | 0.79 | 17.37 | 1.65 | 2 | no data |
|  | **Europe** | 0.97 | 0.81 | 1.15 | -0.41 | 17 | 0.684 |
|  | **North America** | 1.21 | 0.87 | 1.68 | 1.10 | 11 | 0.27 |
|  | **Oceania** | 0.98 | 0.90 | 1.06 | 0.58 | 2 | no data |
|  | **South America** | 0.24 | 0.09 | 0.67 | -2.73 | 6 | ≤0.001 |
| **Landuse** | **Arable** | 1.03 | 0.91 | 1.17 | 0.44 | 35 | 0.662 |
|  | **Grassland** |  |  |  |  | 0 | no data |
|  | **Orchard** | 0.52 | 0.29 | 0.92 | -2.24 | 5 | 0.001 |
| **Plant life cycle** | **Annual** | 1.03 | 0.91 | 1.16 | 0.39 | 36 | 0.698 |
|  | **Perennial** | 0.42 | 0.20 | 0.90 | -2.23 | 4 | 0.001 |
| **Set up** | **Field/Farm** | 0.96 | 0.76 | 1.21 | -0.35 | 18 | 0.135 |
|  | **Longterm** | 0.95 | 0.82 | 1.11 | -0.62 | 22 | 0.533 |
| **Time since conversion** | **4-10 years** | 0.90 | 0.78 | 1.04 | -1.40 | 30 | 0.021 |
|  | **<10 years** | 1.18 | 0.70 | 2.01 | 0.62 | 5 | 0.535 |
|  | **3 years** | 0.94 | 0.61 | 1.47 | -0.26 | 3 | 0.794 |
| **Clay content** | **0-15%** | 0.97 | 0.46 | 2.07 | -0.07 | 3 | 0.946 |
|  | **16-40%** | 0.93 | 0.72 | 1.19 | -0.31 | 16 | 0.148 |
|  | **41-100%** |  |  |  |  | 0 |  |
| **Croprotation** | **Different** | 0.86 | 0.67 | 1.11 | -1.37 | 23 | 0.059 |
|  | **Similar** | 1.09 | 0.91 | 1.32 | 0.91 | 13 | 0.363 |
| **Legumes in Croprotation in:** | **no** | 1.07 | 0.83 | 1.39 | 0.55 | 5 | 0.493 |
|  | **ORG + CON** | 1.00 | 0.87 | 1.16 | 0.02 | 22 | 0.985 |
|  | **ORG** | 0.74 | 0.48 | 1.15 | -1.35 | 13 | 0.093 |
|  | **CON** |  |  |  |  | 0 |  |
| **Oranic Inputs in:** | **no** | 1.22 | 0.95 | 1.57 | 1.58 | 4 | 0.115 |
|  | **ORG + CON** | 0.83 | 0.65 | 1.06 | -1.49 | 7 | 0.137 |
|  | **ORG** | 0.92 | 0.74 | 1.15 | -0.71 | 27 | 0.140 |
|  | **CON** |  |  |  |  | 0 |  |
| **Use of synthetic pesticides** | **CON** | 1.03 | 0.81 | 1.31 | 0.27 | 18 | 0.461 |
|  | **no** | 0.77 | 0.51 | 1.15 | -1.29 | 3 | 0.197 |
|  |  |  |  |  |  |  |  |
|  |  | **Protease activity** | | | | | |
|  |  | **Point** | **CI Lower** | **CI Upper** | **Z-value** | **n** | **p-value** |
| **Full dataset** |  | 1.84 | 1.63 | 2.08 | 9.81 | 7 | ≤0.001 |
| **Climate zone (Köppen)** | **A** |  |  |  |  | 0 |  |
|  | **B** |  |  |  |  | 0 |  |
|  | **C** | 1.84 | 1.63 | 2.08 | 9.81 | 7 | ≤0.001 |
|  | **D** |  |  |  |  | 0 |  |
| **Continent** | **Africa** |  |  |  |  | 0 |  |
|  | **Asia** | 1.87 | 1.45 | 2.42 | 4.79 | 3 | ≤0.001 |
|  | **Europe** | 1.91 | 1.76 | 2.08 | 14.92 | 3 | ≤0.001 |
|  | **North America** | 1.16 | 0.80 | 1.70 | 0.78 | 1 | no data |
|  | **Oceania** |  |  |  |  | 0 |  |
|  | **South America** |  |  |  |  | 0 |  |
| **Landuse** | **Arable** | 2.04 | 1.78 | 2.34 | 10.29 | 2 | no data |
|  | **Grassland** |  |  |  |  | 0 |  |
|  | **Orchard** | 1.74 | 1.47 | 2.07 | 6.28 | 5 | ≤0.001 |
| **Plant life cycle** | **Annual** | 1.91 | 1.76 | 2.08 | 14.92 | 3 | ≤0.001 |
|  | **Perennial** | 1.70 | 1.30 | 2.22 | 3.85 | 4 | ≤0.001 |
| **Set up** | **Field/Farm** | 1.70 | 1.30 | 2.22 | 3.85 | 4 | ≤0.001 |
|  | **Longterm** | 1.91 | 1.76 | 2.08 | 14.92 | 3 | ≤0.001 |
| **Time since conversion** | **4-10 years** | 1.84 | 1.63 | 2.08 | 9.81 | 7 | ≤0.001 |
|  | **<10 years** |  |  |  |  | 0 |  |
|  | **3 years** |  |  |  |  | 0 |  |
| **Clay content** | **0-15%** | 1.70 | 1.30 | 2.22 | 3.85 | 4 | ≤0.001 |
|  | **16-40%** | 1.91 | 1.76 | 2.08 | 14.92 | 3 | ≤0.001 |
|  | **41-100%** |  |  |  |  | 0 |  |
| **Croprotation** | **Different** |  |  |  |  | 0 |  |
|  | **Similar** | 1.91 | 1.73 | 2.10 | 13.20 | 6 | ≤0.001 |
| **Legumes in Croprotation in:** | **no** | 1.90 | 1.59 | 2.28 | 7.04 | 4 | ≤0.001 |
|  | **ORG + CON** | 1.76 | 1.42 | 2.17 | 5.27 | 3 | ≤0.001 |
|  | **ORG** |  |  |  |  | 0 |  |
|  | **CON** |  |  |  |  | 0 |  |
| **Oranic Inputs in:** | **no** |  |  |  |  | 0 |  |
|  | **ORG + CON** | 1.16 | 0.80 | 1.70 | 0.78 | 1 | no data |
|  | **ORG** | 1.91 | 1.73 | 2.10 | 13.20 | 6 | ≤0.001 |
|  | **CON** |  |  |  |  | 0 |  |
| **Use of synthetic pesticides** | **CON** | 1.77 | 1.43 | 2.18 | 5.34 | 5 | ≤0.001 |
|  | **no** | 1.91 | 1.72 | 2.12 | 11.99 | 2 | no data |
|  |  |  |  |  |  |  |  |
|  |  |  |  |  |  |  |  |
|  |  | **Urease activity** | | | | | |
|  |  | **Point** | **CI Lower** | **CI Upper** | **Z-value** | **n** | **p-value** |
| **Full dataset** |  | 1.29 | 1.18 | 1.41 | 5.50 | 18 | ≤0.001 |
| **Climate zone (Köppen)** | **A** | 0.75 | 0.61 | 0.91 | -2.96 | 1 | no data |
|  | **B** | 1.87 | 1.57 | 2.24 | 6.96 | 1 | no data |
|  | **C** | 1.33 | 1.18 | 1.51 | 4.59 | 16 | ≤0.001 |
|  | **D** |  |  |  |  | 0 |  |
| **Continent** | **Africa** |  |  |  |  | 0 |  |
|  | **Asia** | 1.34 | 1.00 | 1.81 | 1.96 | 6 | 0.050 |
|  | **Europe** | 1.64 | 1.53 | 1.75 | 14.54 | 5 | ≤0.001 |
|  | **North America** | 1.87 | 1.57 | 2.24 | 6.96 | 1 | no data |
|  | **Oceania** | 1.10 | 0.96 | 1.25 | 1.39 | 6 | n.s. |
|  | **South America** |  |  |  |  | 0 |  |
| **Landuse** | **Arable** | 1.39 | 1.20 | 1.60 | 4.45 | 13 | ≤0.001 |
|  | **Grassland** |  |  |  |  | 0 |  |
|  | **Orchard** | 1.16 | 0.90 | 1.49 | 1.15 | 15 | n.s. |
| **Plant life cycle** | **Annual** | 1.38 | 1.20 | 1.59 | 4.56 | 4 | ≤0.001 |
|  | **Perennial** | 1.13 | 0.85 | 1.51 | 0.86 | 14 | n.s. |
| **Set up** | **Field/Farm** | 1.23 | 1.05 | 1.44 | 2.62 | 13 | 0.009 |
|  | **Longterm** | 1.64 | 1.53 | 1.75 | 14.54 | 5 | ≤0.001 |
| **Time since conversion** | **4-10 years** | 1.27 | 1.11 | 1.45 | 3.43 | 13 | ≤0.001 |
|  | **<10 years** | 1.18 | 0.48 | 2.92 | 0.37 | 2 | no data |
|  | **3 years** | 1.63 | 1.50 | 1.78 | 11.63 | 1 | no data |
| **Clay content** | **0-15%** | 1.31 | 1.16 | 1.47 | 4.35 | 3 | ≤0.001 |
|  | **16-40%** | 1.49 | 1.22 | 1.82 | 3.94 | 8 | ≤0.001 |
|  | **41-100%** |  |  |  |  | 0 |  |
| **Croprotation** | **Different** | 1.22 | 1.00 | 1.49 | 1.96 | 10 | n.s. |
|  | **Similar** | 1.52 | 1.40 | 1.66 | 9.52 | 8 | ≤0.001 |
| **Legumes in Croprotation in:** | **no** | 1.34 | 1.15 | 1.55 | 3.74 | 8 | ≤0.001 |
|  | **ORG + CON** | 1.22 | 1.02 | 1.46 | 2.17 | 9 | 0.030 |
|  | **ORG** | 7.00 | 3.29 | 14.89 | 5.05 | 1 | no data |
|  | **CON** |  |  |  |  | 0 |  |
| **Oranic Inputs in:** | **no** |  |  |  |  | 0 |  |
|  | **ORG + CON** | 1.64 | 1.55 | 1.74 | 16.86 | 3 | ≤0.001 |
|  | **ORG** | 1.25 | 1.08 | 1.46 | 2.94 | 15 | 0.003 |
|  | **CON** |  |  |  |  | 0 |  |
| **Use of synthetic pesticides** | **CON** | 1.41 | 1.20 | 1.66 | 4.16 | 9 | ≤0.001 |
|  | **no** | 1.98 | 0.67 | 5.81 | 1.24 | 2 | no data |
|  |  |  |  |  |  |  |  |
