# Supplementary material for: Organic farming enhances soil microbial abundance and activity—A meta-analysis and meta-regression
Source: PLoS One. 2017 Jul 12;12(7):e0180442. doi: 10.1371/journal.pone.0180442 (PMC5507504; doi:10.1371/journal.pone.0180442)
Supplement: S5 Table — Random effects model with Knapp-Hartung adjustments were applied with the listed covariates. All computational procedures were done using logarithmized data to obtain a normally distributed dataset. (DOCX) [file pone.0180442.s006.docx]

|  | **Covariate** | **Coefficient** | **SE** | **Lower 95% CI** | **Upper 95% CI** | **Z-value** | **2-sided p-value** |  |
| --- | --- | --- | --- | --- | --- | --- | --- | --- |
|  |  |  |  |  |  |  |  | Test of the model |
| Microbial biomass carbon | **Intercept** | **-0,1748** | **0,4746** | **-1,1051** | **0,7555** | **-0,37** | **0.713** | Q = 40,60, df = 6, p≤0.001 |
|  | **SOC mean** | **-0,1554** | **0,0769** | **-0,306** | **-0,0047** | **-2,02** | **0.043** | Goodness of fit |
|  | **SOC difference** | **0,2826** | **0,1047** | **0,0773** | **0,4879** | **2,7** | **0.007** | Tau² = 0,0537, Tau = 0,2317, I² = 63,33%, Q = 136,34, df = 50, p≤0.001 |
|  | **TN mean** | **1,1639** | **0,8917** | **-0,5839** | **2,9116** | **1,31** | **0.192** | Total between-study variance |
|  | **TN difference** | **1,295** | **0,7943** | **-0,2617** | **2,8518** | **1,63** | **0.103** | Tau² = 0,0879, Tau = 0,2964, I² = 80,68%, Q = 289,83, df = 56, p≤0.001 |
|  | **pH mean** | **0,0665** | **0,0638** | **-0,0585** | **0,1914** | **1,04** | **0.297** | Proportion of total between-study variance |
|  | **pH difference** | **-0,1405** | **0,1182** | **-0,3722** | **0,0912** | **-1,19** | **0.235** | R² analog = 0,39 |
|  |  |  |  |  |  |  |  | Test of the model |
| Microbial biomass nitrogen | **Intercept** | **-3,9859** | **2,5989** | **-9,0797** | **1,1078** | **-1,53** | **0.125** | Q = 17,09, df = 6, p≤0.001 |
|  | **SOC mean** | **-0,3288** | **0,2877** | **-0,8927** | **0,235** | **-1,14** | **0.253** | Goodness of fit |
|  | **SOC difference** | **1,1396** | **0,672** | **-0,1776** | **2,4568** | **1,7** | **0.090** | Tau² = 0,2147, Tau = 0,4633, I² = 93,26%, Q = 177,92, df = 12, p≤0.001 |
|  | **TN mean** | **4,1707** | **3,7379** | **-3,1554** | **11,4968** | **1,12** | **0.265** | Total between-study variance |
|  | **TN difference** | **-13,5302** | **8,7579** | **-30,6955** | **3,635** | **-1,54** | **0.122** | Tau² = 0,3269, Tau = 0,5717, I² = 98,12%, Q = 959,18, df = 18, p≤0.001 |
|  | **pH mean** | **0,6074** | **0,3726** | **-0,1228** | **1,3377** | **1,63** | **0.103** | Proportion of total between-study variance |
|  | **pH difference** | **0,1843** | **0,4774** | **-0,7514** | **1,1201** | **0,39** | **0.699** | R² analog = 0,34 |
|  |  |  |  |  |  |  |  | Test of the model |
| Total PLFA | **Intercept** | **-0,252** | **1,6753** | **-3,5354** | **3,0315** | **-0,15** | **0.880** | Q = 4,72, df = 6, p = 0,5808 |
|  | **SOC mean** | **-0,569** | **0,6375** | **-1,8184** | **0,6804** | **-0,89** | **0.372** | Goodness of fit |
|  | **SOC difference** | **0,0191** | **0,7453** | **-1,4417** | **1,4799** | **0,03** | **0.980** | Tau² = 0,0739, Tau = 0,2718, I² = 88,82%, Q = 53,69, df = 6, p≤0.001 |
|  | **TN mean** | **6,0047** | **5,4949** | **-4,7652** | **16,7745** | **1,09** | **0.275** | Total between-study variance |
|  | **TN difference** | **1,0868** | **7,1558** | **-12,9384** | **15,1119** | **0,15** | **0.879** | Tau² = 0,0662, Tau = 0,2574, I² = 92,65%, Q = 163,23, df = 12, p≤0.001 |
|  | **pH mean** | **0,0792** | **0,219** | **-0,3501** | **0,5084** | **0,36** | **0.718** | Proportion of total between-study variance |
|  | **pH difference** | **-0,0345** | **0,3463** | **-0,7132** | **0,6443** | **-0,1** | **0.921** | R² analog = 0,00 (computed value is -0,12) |
|  |  |  |  |  |  |  |  | Test of the model |
| Dehydrogenase activity | **Intercept** | **-0,151** | **0,9859** | **-2,0833** | **1,7812** | **-0,15** | **0.878** | Q = 15,23, df = 6, p = 0,018 |
|  | **SOC mean** | **-0,0718** | **0,0679** | **-0,2049** | **0,0612** | **-1,06** | **0.290** | Goodness of fit |
|  | **SOC difference** | **0,6569** | **0,495** | **-0,3133** | **1,6271** | **1,33** | **0.185** | Tau² = 0,0427, Tau = 0,2066, I² = 51,49%, Q = 41,23, df = 20, p≤0.001 |
|  | **TN mean** | **0,9089** | **1,4388** | **-1,9111** | **3,7288** | **0,63** | **0.528** | Total between-study variance |
|  | **TN difference** | **-2,2705** | **4,8791** | **-11,8333** | **7,2924** | **-0,47** | **0.642** | Tau² = 0,0577, Tau = 0,2402, I² = 58,60%, Q = 62,81, df = 26, p≤0.001 |
|  | **pH mean** | **0,075** | **0,1288** | **-0,1774** | **0,3273** | **0,58** | **0.561** | Proportion of total between-study variance |
|  | **pH difference** | **0,4606** | **0,1525** | **0,1617** | **0,7595** | **3,02** | **0.003** | R² analog = 0,26 |
|  |  |  |  |  |  |  |  | Test of the model |
| Metabolic quotient | **Intercept** | **-1,8068** | **1,868** | **-5,4679** | **1,8544** | **-0,97** | **0.333** | Q = 10,32, df = 6, p = 0,112 |
|  | **SOC mean** | **-0,3487** | **0,1991** | **-0,7388** | **0,0415** | **-1,75** | **0.080** | Goodness of fit |
|  | **SOC difference** | **-0,4614** | **0,3998** | **-1,2449** | **0,3221** | **-1,15** | **0.248** | Tau² = 0,0970, Tau = 0,3115, I² = 84,83%, Q = 79,12, df = 12, p≤0.001 |
|  | **TN mean** | **5,2916** | **2,3507** | **0,6844** | **9,8988** | **2,25** | **0.024** | Total between-study variance |
|  | **TN difference** | **3,0339** | **8,0155** | **-12,6761** | **18,744** | **0,38** | **0.705** | Tau² = 0,0852, Tau = 0,2918, I² = 87,47%, Q = 143,62, df = 18, p≤0.001 |
|  | **pH mean** | **0,2056** | **0,2867** | **-0,3564** | **0,7676** | **0,72** | **0.473** | Proportion of total between-study variance |
|  | **pH difference** | **0,0806** | **0,26** | **-0,429** | **0,5902** | **0,31** | **0.757** | R² analog = 0,00 (computed value is -0,14) |
|  |  |  |  |  |  |  |  | Test of the model |
| Urease activity | **Intercept** | **0,7464** | **0,6735** | **-0,5737** | **2,0665** | **1,11** | **0.268** | Q = 29,11, df = 6, p≤0.001 |
|  | **SOC mean** | **0,0943** | **0,1019** | **-0,1054** | **0,294** | **0,93** | **0.355** | Goodness of fit |
|  | **SOC difference** | **0,2646** | **0,2346** | **-0,1953** | **0,7244** | **1,13** | **0.260** | Tau² = 0,0201, Tau = 0,1419, I² = 71,59%, Q = 38,71, df = 11, p≤0.001 |
|  | **TN mean** | **-2,8305** | **1,3517** | **-5,4797** | **-0,1813** | **-2,09** | **0.036** | Total between-study variance |
|  | **TN difference** | **1,6318** | **2,3126** | **-2,9008** | **6,1645** | **0,71** | **0.480** | Tau² = 0,0613, Tau = 0,2476, I² = 90,46%, Q = 178,20, df = 17, p≤0.001 |
|  | **pH mean** | **-0,0239** | **0,0824** | **-0,1854** | **0,1376** | **-0,29** | **0.772** | Proportion of total between-study variance |
|  | **pH difference** | **0,357** | **0,1518** | **0,0595** | **0,6546** | **2,35** | **0.019** | R² analog = 0,67 |
