# Supplementary material for: Organic farming enhances soil microbial abundance and activity—A meta-analysis and meta-regression
Source: PLoS One. 2017 Jul 12;12(7):e0180442. doi: 10.1371/journal.pone.0180442 (PMC5507504; doi:10.1371/journal.pone.0180442)
Supplement: S7 Table — (DOCX) [file pone.0180442.s008.docx]

|  | LnRR | VarLnRR | MAP | MAT | pH mean | Clay mean | SOC mean | TN mean | Organic Inputs | Legumes in CR | Synthetic pesticide | CR | Clay | Plant life cycle | Set up | Duration | Landuse | | Climate Zone 1 |
| --- | --- | --- | --- | --- | --- | --- | --- | --- | --- | --- | --- | --- | --- | --- | --- | --- | --- | --- | --- |
| Amaral et al. 2012 | 1.100 | 0.083 |  |  | 5.3 | 20.6 | 0.53 |  | ORG only | ORG only | CON only | Different | Middle | Perennial | e |  | Orchard | Oceania | Cfa |
| Amaral et al. 2012 | 1.280 | 0.113 |  |  | 5.3 | 20.6 | 0.53 |  | ORG only | ORG only | CON only | Different | Middle | Perennial | e |  | Orchard | South America | Cfa |
| Andrews et al. 2002 | 0.812 | 0.055 | 1958 | 17.3 |  |  | 1.80 |  | ORG only | CON only | CON only | Different | Middle | Annual | fa | Middle | Arable | North America | Csa |
| Anonymous, Unpublsihed | 0.359 | 0.041 | 1950 | 20.0 | 6.1 |  | 2.55 | 0.181 | ORG and CON | ORG and CON | CON only | Different |  | Annual | e | Middle | Arable | Africa | Aw |
| Anonymous, Unpublsihed | -0.132 | 0.037 | 1000 | 20.0 | 6.4 |  | 2.14 | 0.248 | ORG and CON | ORG and CON | CON only | Different |  | Annual | e | Middle | Arable | Africa | Cfb |
| Bolton Jr et al. 1985 | 0.136 | 0.023 |  |  | 5.8 | . | 1.03 | 0.112 | ORG only | ORG and CON | . | Different |  | Annual | f | Old | Arable | North America | BSk |
| Bossio et al. 1998 | 0.111 | 0.032 | 635 |  |  |  |  |  | ORG only | ORG only | CON only | Different |  | Annual | e | Middle | Arable | North America | Csb |
| Bossio et al. 1998 | -0.099 | 0.026 | 635 |  |  |  |  |  | none | ORG and CON | CON only | Different |  | Annual | e | Middle | Arable | North America | Csb |
| Burger et al. 2005 | 0.537 | 0.280 | 499 | 16.2 | 6.7 | 22.5 | 1.27 | 0.120 | ORG only | ORG only | . | Different | Middle | Annual | e | Old | Arable | North America | Csa |
| Carey et al. 2009 | 0.182 | 0.005 | 1735 | 14.7 | 6.6 |  | 5.40 | 0.450 |  | ORG and CON |  |  |  | Annual | f | Middle | Arable | Oceania | Cfb |
| Carey et al. 2009 | 0.106 | 0.005 | 1735 | 14.7 | 6.6 |  | 5.65 | 0.470 |  | ORG and CON |  |  |  | Annual | f | Middle | Arable | Oceania | Cfb |
| Carpenter-Boggs et al. 2000 | 0.405 | 0.053 | 550 |  |  |  | 2.03 |  | ORG only | ORG and CON |  | Similar | Light | Annual | e |  | Arable | North America | BSk |
| Carpenter-Boggs et al. 2000 | 0.365 | 0.051 | 550 |  |  |  | 2.03 |  | ORG only | ORG and CON |  | Similar |  | Annual | e |  | Arable | North America | BSk |
| Fraser et al. 1989 | 0.000 | 0.045 | 680 | 11.0 | 7.0 |  | 1.88 |  | ORG only | ORG and CON | CON only | Similar |  | Annual | e | Middle | Arable | North America | Dfa |
| Freitas et al. 2011 | 0.907 | 0.037 | 450 | 26.0 | 7.0 | 30.0 | 0.91 |  | ORG only | none | CON only | Similar | Middle | Perennial | f | Young | Orchard | South America | BSh |
| Gajda and Martyniuk, 2004 | 0.196 | 0.062 |  |  | 6.6 |  | 0.78 |  | ORG only | ORG only | CON only | Different |  | Annual | e | Middle | Arable | Europe | Dfb |
| Gajda and Martyniuk, 2004 | 0.441 | 0.072 |  |  | 6.5 |  | 0.82 |  | ORG only | ORG only | CON only | Different |  | Annual | e | Middle | Arable | Europe | Dfb |
| Ge et al. 2011 | 1.346 | 0.068 | 1255 | 17.5 | 8.2 | 20.0 | 1.21 | 0.155 | ORG only | none | CON only | Different | Middle | Annual | e |  | Arable | Asia | Cfa |
| Ge et al. 2011 | 1.620 | 0.048 | 1255 | 17.5 | 7.8 | 20.0 | 1.75 | 0.252 | ORG only | ORG only | CON only | Different | Middle | Annual | e |  | Arable | Asia | Cfa |
| Ge et al. 2013 | 1.335 | 0.026 | 1255 | 17.5 | 7.9 | 21.0 | 1.52 | 0.222 | ORG only | none | CON only | Different | Middle | Annual | e | Middle | Arable | Asia | Cfa |
| Ge et al. 2013 | 1.315 | 0.025 | 1255 | 17.5 | 8.3 | 21.0 | 1.11 | 0.143 | ORG only | none | CON only | Different | Middle | Annual | e | Middle | Arable | Asia | Cfa |
| Glover et al. 2000 | 0.000 | 0.045 |  |  | 6.4 |  | 1.53 | 0.169 | ORG and CON | none | CON only |  |  | Perennial | e | Middle | Orchard | North America | BWk |
| Glover et al. 2000 | 0.114 | 0.047 |  |  | 6.5 |  | 1.40 | 0.156 | ORG only | none | CON only |  |  | Perennial | e | Middle | Orchard | North America | BWk |
| Gonzales-Perez et al. 2015 | 0.190 | 0.006 | 1255 | 17.5 | 7.0 | 21.0 | 2.27 | 0.280 | ORG only | ORG and CON | CON only |  | Middle | Annual | e | Old | Arable | Asia | Cfa |
| Gunapala et al. 1998 | 0.336 | 0.050 | 635 |  |  |  |  |  | ORG only | ORG only | CON only | Different |  | Annual | e |  | Arable | North America | Csa |
| Hartmann et al. 2006 | 0.655 | 0.064 | 785 | 9.5 | 6.5 | 15.0 | 1.36 | 0.161 | ORG only | ORG and CON | CON only | Similar | Light | Annual | e | Old | Arable | Europe | Cfb |
| Hartmann et al. 2006 | 0.262 | 0.022 | 785 | 9.5 | 6.5 | 15.0 | 1.37 | 0.159 | ORG and CON | ORG and CON | CON only | Similar | Light | Annual | e | Old | Arable | Europe | Cfb |
| Hartmann et al. 2006 | 0.699 | 0.101 | 785 | 9.5 | 6.5 | 15.0 | 1.32 | 0.175 | ORG only | ORG and CON | CON only | Similar | Light | Annual | e | Old | Arable | Europe | Cfb |
| Hartmann et al. 2006 | 0.306 | 0.043 | 785 | 9.5 | 6.5 | 15.0 | 1.33 | 0.173 | ORG and CON | ORG and CON | CON only | Similar | Light | Annual | e | Old | Arable | Europe | Cfb |
| Hartmann et al. 2006 | 0.403 | 0.037 | 785 | 9.5 | 6.5 | 15.0 | 1.36 | 0.161 | ORG only | ORG and CON | CON only | Similar | Light | Annual | e | Old | Arable | Europe | Cfb |
| Hartmann et al. 2006 | 0.160 | 0.015 | 785 | 9.5 | 6.5 | 15.0 | 1.37 | 0.159 | ORG and CON | ORG and CON | CON only | Similar | Light | Annual | e | Old | Arable | Europe | Cfb |
| Hartmann et al. 2006 | 0.547 | 0.132 | 785 | 9.5 | 6.5 | 15.0 | 1.32 | 0.175 | ORG only | ORG and CON | CON only | Similar | Light | Annual | e | Old | Arable | Europe | Cfb |
| Hartmann et al. 2006 | 0.304 | 0.083 | 785 | 9.5 | 6.5 | 15.0 | 1.33 | 0.173 | ORG and CON | ORG and CON | CON only | Similar | Light | Annual | e | Old | Arable | Europe | Cfb |
| Heinze et al. 2010 | 0.091 | 0.045 | 590 | 9.5 | 6.6 | 5.0 | 0.85 | 0.079 | ORG only | ORG and CON |  | Similar |  | Annual | e | Old | Arable | Europe | Cfb |
| Heinze et al. 2010 | 0.091 | 0.045 | 590 | 9.5 | 6.6 | 5.0 | 0.86 | 0.080 | ORG only | ORG and CON |  | Similar | Light | Annual | e | Old | Arable | Europe | Cfb |
| Islam and Weil 2000 | 0.424 | 0.054 |  |  | 6.1 | 27.0 | 2.35 | 0.313 | ORG only | ORG and CON |  | Different | Middle | Annual | e | Old | Arable | North America | Dfa |
| Islam and Weil 2000 | -0.033 | 0.045 |  |  | 6.1 | 27.1 | 2.39 | 0.308 | none | ORG and CON |  | Different | Middle | Annual | e | Old | Arable | North America | Dfa |
| Jacinthe et al. 2011 | 0.671 | 0.091 | 205 | 20.0 | 7.8 |  | 0.90 |  | ORG only | ORG only |  | Different |  | Annual | f | Young | Arable | South America | BSh |
| Jacinthe et al. 2011 | 0.241 | 0.064 | 205 | 20.0 | 7.7 |  | 0.84 |  | ORG only | ORG only |  | Different |  | Annual | f | Middle | Arable | South America | BSh |
| Jacinthe et al. 2011 | 0.788 | 0.106 | 205 | 20.0 | 7.8 |  | 0.75 |  | ORG only | ORG only |  | Different |  | Annual | f | Middle | Arable | South America | BSh |
| Kramer et al. 2006 | 0.198 | 0.047 |  |  |  | 6.4 | 1.89 | 0.186 | ORG and CON | none | CON only | Similar | Light | Perennial | f |  | Orchard | North America | BWk |
| Kramer et al. 2006 | 0.360 | 0.043 |  |  |  | 6.5 | 1.64 | 0.160 | ORG only | none | CON only | Similar | Light | Perennial | f |  | Orchard | North America | BWk |
| Lagomarsino et al. 2009 | 0.103 | 0.027 |  |  |  |  | 2.10 |  | none | ORG and CON |  | Different |  | Annual | e | Middle | Arable | Europe | Csa |
| Lagomarsino et al. 2009 | 0.818 | 0.249 |  |  |  |  | 1.95 |  | ORG only | ORG and CON |  | Different |  | Annual | e | Middle | Arable | Europe | Csa |
| Lagomarsino et al. 2009 | 0.485 | 0.105 |  |  |  |  | 2.15 |  | ORG only | ORG and CON |  | Different |  | Annual | e | Middle | Arable | Europe | Csa |
| Larsen et al. 2014 | 0.505 | 0.612 | 1084 | 12.6 |  |  | 1.26 |  |  | ORG only | CON only |  |  | Annual | e | Old | Arable | North America | Cfb |
| Larsen et al. 2014 | 1.039 | 1.496 | 1084 | 12.6 |  |  | 1.06 |  |  | ORG only | CON only |  |  | Annual | e | Old | Arable | North America | Cfb |
| Larsen et al. 2014 | -0.284 | 0.588 | 1084 | 12.6 |  |  | 1.01 |  |  | ORG only | CON only |  |  | Annual | e | Old | Arable | North America | Cfb |
| Larsen et al. 2014 | 0.250 | 1.033 | 1084 | 12.6 |  |  | 0.81 |  |  | ORG only | CON only |  |  | Annual | e | Old | Arable | North America | Cfb |
| Liebig and Doran 1999 | 0.226 | 0.063 |  |  | 6.1 |  |  |  | ORG only | ORG only |  | Different |  | Annual | f | Middle | Arable | North America | Dfa |
| Liebig and Doran 1999 | 0.513 | 0.077 |  |  | 5.7 |  |  |  | none | ORG and CON |  | Different |  | Annual | f | Old | Arable | North America | Dfa |
| Liebig and Doran 1999 | 0.600 | 0.084 |  |  | 6.4 |  |  |  | ORG only | ORG and CON |  | Different |  | Annual | f | Middle | Arable | North America | Dfa |
| Liebig and Doran 1999 | 0.665 | 0.091 |  |  | 7.4 |  |  |  | ORG only | ORG only |  | Different |  | Annual | f | Old | Arable | North America | Dfa |
| Liebig and Doran 1999 | -0.048 | 0.060 |  |  | 6.8 |  |  |  | ORG only | ORG only |  | Different |  | Annual | f | Old | Arable | North America | Dfa |
| Liu et al. 2007 | 0.494 | 0.076 |  |  |  |  | 1.63 | 0.277 | ORG only | ORG only | no | Different |  | Annual | f | Old | Arable | North America | Cfa |
| Liu et al. 2007 | 1.321 | 0.153 |  |  |  |  | 1.18 | 0.232 | ORG only | ORG only | no | Different |  | Annual | f | Old | Arable | North America | Cfa |
| Marinari et al. 2006 | 0.533 | 0.039 |  |  | 7.6 | 20.0 | 0.96 | 0.100 | ORG only | none | CON only | Similar | Middle | Annual | f | Middle | Arable | Europe | Csa |
| Marinari et al. 2010 | -0.163 | 0.021 |  |  |  |  | 1.19 | 0.135 | ORG only | ORG only | CON only | Similar |  | Annual | e | Middle | Arable | Europe | Csa |
| Mazzoncini et al. 2010 | 0.003 | 0.060 | 700 |  |  |  | 0.87 | 0.105 | ORG only | ORG and CON |  | Similar |  | Annual | e | Middle | Arable | Europe | Csa |
| Melero et al. 2006 | 1.230 | 0.064 |  |  | 8.4 | 23.0 | 1.52 | 0.175 | ORG only | ORG and CON | none | Similar | Middle | Annual | e | Middle | Arable | Europe | Csa |
| Melero et al. 2006 | 0.292 | 0.024 |  |  | 7.8 | 23.0 | 1.46 | 0.155 | ORG only | ORG and CON | none | Similar | Middle | Annual | e | Middle | Orchard | Europe | Csa |
| Migliorini et al. 2014 | -0.210 | 0.047 | 770 | 14.1 | 8.3 | 33.4 | 1.03 | 0.117 | ORG only | ORG only | CON only | Different | Middle | Annual | e | Old | Arable | Europe | Csa |
| Migliorini et al. 2014 | -0.197 | 0.047 | 770 | 14.1 | 8.3 | 33.6 | 1.05 | 0.113 | ORG only | ORG only | CON only | Different | Middle | Annual | e | Middle | Arable | Europe | Csa |
| Moeskops et al. 2010 | 0.313 | 0.147 | 3035 | 21.3 | 4.5 |  | 3.07 | 0.330 | ORG and CON | none | CON only | Similar |  | Annual | f | Middle | Arable | Asia | Af |
| Moeskops et al. 2010 | 0.361 | 0.048 | 3035 | 21.3 | 4.5 |  | 3.05 | 0.350 | ORG and CON | none | CON only | Similar |  | Annual | f | Middle | Arable | Asia | Af |
| Moeskops et al. 2010 | 0.794 | 0.139 | 3035 | 21.3 | 5.0 |  | 4.80 | 0.505 | ORG and CON | none | CON only | Similar |  | Annual | f | Middle | Arable | Asia | Af |
| Moeskops et al. 2010 | 0.323 | 0.076 | 3035 | 21.3 | 5.6 |  | 5.05 | 0.530 | ORG and CON | none | CON only | Similar |  | Annual | f | Middle | Arable | Asia | Af |
| Moeskops et al. 2010 | 0.088 | 0.074 | 3035 | 21.3 | 4.8 |  | 3.59 | 0.410 | ORG and CON | none | CON only | Similar |  | Annual | f | Young | Arable | Asia | Af |
| Moeskops et al. 2010 6 | 0.288 | 0.072 | 3035 | 21.3 | 4.9 |  | 3.17 | 0.365 | ORG and CON | none | CON only | Similar |  | Annual | f | Young | Arable | Asia | Af |
| Monokrousos et al. 2006 | 0.078 | 0.008 | 506 |  | 7.7 | 26.1 | 1.43 | 0.067 | ORG and CON | none | CON only | Similar | Middle | Annual | ff | Middle | Arable | Europe | Cfa |
| Monokrousos et al. 2006 | 0.051 | 0.013 | 506 |  | 7.9 | 29.6 | 1.61 | 0.073 | ORG and CON | none | CON only | Similar | Middle | Annual | f | Middle | Arable | Europe | Cfa |
| Monokrousos et al. 2006 | 0.176 | 0.019 | 506 |  | 7.9 | 28.2 | 1.42 | 0.068 | ORG and CON | none | CON only | Similar | Middle | Annual | f | Young | Arable | Europe | Cfa |
| Murata et al. 1997 | 0.142 | 0.054 | 1050 |  |  |  | 3.43 | 0.336 | ORG and CON | ORG and CON |  | Different |  | Annual | f | Middle | Arable | Oceania | Cfb |
| Murata et al. 1997 | -0.341 | 0.004 | 1050 |  |  |  | 3.44 | 0.337 | ORG and CON | ORG and CON |  | Different |  | Perennial | f | Middle | Grassland | Oceania | Cfb |
| Oberholzer et al. 2000 | 0.059 | 0.153 |  |  | 6.5 | 26.5 | 1.30 |  | ORG and CON | ORG and CON | CON only |  | Middle | Annual | f |  | Arable | Europe | Cfb |
| Okur et al. 2009 | 0.390 | 0.070 | 706 | 17.0 | 7.5 | 15.9 | 0.77 | 0.135 | ORG only | none | CON only | Similar | Light | Perennial | f |  | Orchard | Asia | Csa |
| Okur et al. 2009 | 0.322 | 0.066 | 706 | 17.0 | 7.5 | 15.9 | 0.77 | 0.135 | ORG only | none | CON only | Similar | Light | Perennial | f |  | Orchard | Asia | Csa |
| Okur et al. 2009 | 0.229 | 0.063 | 706 | 17.0 | 7.5 | 15.9 | 0.77 | 0.135 | ORG only | none | CON only | Similar | Light | Perennial | f |  | Orchard | Asia | Csa |
| Padmavathy and Poyyamoli 2011 | 0.562 | 0.001 | 1242 | 30.0 | 7.5 |  | 0.90 | 0.267 | ORG only | ORG and CON | CON only | Similar |  | Annual | f | Middle | Arable | Asia | Aw |
| Petersen et al. 1997 | 0.464 | 0.046 | 653 | 8.6 | 6.1 | 17.5 | 1.66 | 0.210 | ORG only |  |  |  | Middle | Annual | f | Old | Arable | Europe |  |
| Petersen et al. 1997 | 0.402 | 0.048 | 653 | 8.6 | 6.0 | 17.4 | 1.66 | 0.215 | ORG only |  |  |  | Middle | Annual | f | Old | Arable | Europe |  |
| Petersen et al. 1997 | 0.239 | 0.048 | 653 | 8.6 | 6.1 | 15.5 | 1.49 | 0.210 | ORG only |  |  |  | Light | Annual | f | Old | Arable | Europe |  |
| Petersen et al. 1997 | 0.298 | 0.045 | 653 | 8.6 | 5.7 | 14.3 | 1.33 | 0.215 | ORG only |  |  |  | Light | Annual | f | Old | Arable | Europe |  |
| Petersen et al. 1997 | 0.323 | 0.026 | 653 | 8.6 | 6.2 | 16.0 | 1.70 | 0.220 | ORG only |  |  |  | Light | Annual | f | Old | Arable | Europe |  |
| Petersen et al. 1997 | 0.261 | 0.027 | 653 | 8.6 | 6.1 | 15.9 | 1.70 | 0.225 | ORG only |  |  |  | Light | Annual | f | Old | Arable | Europe |  |
| Petersen et al. 1997 | 0.098 | 0.026 | 653 | 8.6 | 6.2 | 13.9 | 1.53 | 0.220 | ORG only |  |  |  | Light | Annual | f | Old | Arable | Europe |  |
| Petersen et al. 1997 | 0.157 | 0.025 | 653 | 8.6 | 5.8 | 12.7 | 1.37 | 0.225 | ORG only |  |  |  | Light | Annual | f | Old | Arable | Europe |  |
| Reganold et al. 2010 | 0.953 | 0.034 |  |  | 7.1 | 13.1 | 0.91 | 0.077 | ORG and CON | ORG and CON | CON only |  | Light | Perennial | f |  | Orchard | North America | Csb |
| Schjønning et al. 2002 | 0.292 | 0.003 | 521 | 8.2 | 6.7 | 15.5 | 1.51 |  | ORG and CON | ORG and CON |  | Different | Light | Annual | e | Old | Arable | Europe | Cfb |
| Schjønning et al. 2002 | 0.597 | 0.003 | 521 | 8.2 | 6.6 | 15.0 | 1.42 |  | ORG only | ORG and CON |  | Different | Light | Annual | e | Old | Arable | Europe | Cfb |
| Schjønning et al. 2002 | 0.308 | 0.021 | 521 | 8.2 | 6.9 | 20.5 | 2.15 |  | ORG and CON | none |  | Different | Middle | Annual | e | Old | Arable | Europe | Cfb |
| Schjønning et al. 2002 | 0.528 | 0.070 | 521 | 8.2 | 6.2 | 18.0 | 1.72 |  | ORG only | none |  | Different | Middle | Annual | e | Old | Arable | Europe | Cfb |
| Stark et al. 2008 | -0.217 | 0.049 | 680 | 11.5 | 5.9 |  | 2.35 | 0.230 | ORG and CON | ORG and CON |  | Different |  | Annual | e |  | Arable | Oceania | Cfb |
| Stark et al. 2008 | 0.088 | 0.070 | 680 | 11.5 | 5.9 |  | 2.35 | 0.230 | ORG and CON | ORG and CON |  | Different |  | Annual | e |  | Arable | Oceania | Cfb |
| Wells et al. 2000 | 0.380 | 0.069 | 1300 |  | 5.4 | 15.0 | 1.67 | 0.124 | ORG only | ORG only | CON only | Different | Light | Annual | e |  | Arable | Oceania | Cfa |
| Wells et al. 2000 | 0.186 | 0.062 | 1300 |  | 5.9 | 15.0 | 1.52 | 0.112 | ORG only | ORG and CON | CON only | Different | Light | Annual | e |  | Arable | Oceania | Cfa |
| Wells et al. 2000 | 0.356 | 0.068 | 1300 |  | 5.8 | 15.0 | 1.64 | 0.118 | ORG only | ORG and CON | CON only | Different | Light | Annual | e |  | Arable | Oceania | Cfa |
| Yeates et al. 1997 | 2.782 | 1.182 | 1230 | 9.5 | 5.6 | 49.0 | 5.10 |  | ORG only | ORG and CON |  | Similar | Heavy | Perennial | f |  | Grassland | Europe | Cfb |
| Yeates et al. 1997 | -0.590 | 0.025 | 1040 | 9.6 | 6.0 | 37.6 | 5.85 |  | none | ORG and CON |  | Similar | Middle | Perennial | f |  | Grassland | Europe | Cfb |
| Yeates et al. 1997 | 0.262 | 0.019 | 820 | 9.6 | 6.1 | 28.2 | 5.70 |  | only con | ORG and CON |  | Similar | Middle | Perennial | f |  | Grassland | Europe | Cfb |
| Nmic |  |  |  |  |  |  |  |  |  |  |  |  |  |  |  |  |  |  |  |
| Andrews et al. 2002 | 0.837 | 0.039 | 1958 | 17.3 |  |  | 1.80 |  | ORG only | CON only | CON only |  |  | Annual | fa | Middle | Arable | North America | Csa |
| Anonymous, Unpublsihed | 0.227 | 0.026 | 1950 | 20.0 | 6.1 |  | 2.55 | 0.181 | ORG and CON | ORG and CON | CON only | Different |  | Annual | e | Middle | Arable | Africa | Aw |
| Anonymous, Unpublsihed | -0.197 | 0.026 | 1000 | 20.0 | 6.4 |  | 2.14 | 0.248 | ORG and CON | ORG and CON | CON only | Different |  | Annual | e | Middle | Arable | Africa | Cfb |
| Bossio et al. 1998 | 0.094 | 0.031 | 635 |  |  |  |  |  | ORG only | ORG only | CON only | Different |  | Annual | e | Middle | Arable | North America | Csb |
| Bossio et al. 1998 | -0.116 | 0.029 | 635 |  |  |  |  |  | none | ORG and CON | CON only | Different |  | Annual | e | Middle | Arable | North America | Csb |
| Carey et al. 2009 | 0.358 | 0.004 | 1735 | 14.7 | 6.6 |  | 5.40 | 0.450 |  | ORG and CON |  |  |  | Annual | f | Middle | Arable | Oceania | Cfb |
| Carey et al. 2009 | 0.215 | 0.004 | 1735 | 14.7 | 6.6 |  | 5.65 | 0.470 |  | ORG and CON |  |  |  | Annual | f | Middle | Arable | Oceania | Cfb |
| Chirinda et al. 2010 | 0.125 | 0.027 |  |  |  |  | 2.15 |  | ORG only | ORG and CON |  | Similar |  | Annual | e | Middle | Arable | Europe | Dfb |
| Chirinda et al. 2010 | 0.210 | 0.052 |  |  |  |  | 2.10 |  | none | ORG and CON |  | Similar |  | Annual | e | Middle | Arable | Europe | Dfb |
| Chirinda et al. 2010 | 0.210 | 0.018 |  |  |  |  | 1.95 |  | ORG only | ORG and CON |  | Similar |  | Annual | e | Middle | Arable | Europe | Dfb |
| Chirinda et al. 2010 | 0.336 | 0.043 |  |  |  |  | 2.15 |  | ORG only | ORG and CON |  | Different |  | Annual | e | Middle | Arable | Europe | Dfb |
| Chirinda et al. 2010 | 0.105 | 0.009 |  |  |  |  | 2.15 |  | ORG only | ORG and CON |  | Similar |  | Annual | e | Middle | Arable | Europe | Dfb |
| Chirinda et al. 2010 | 0.201 | 0.041 |  |  |  |  | 2.10 |  | none | ORG and CON |  | Similar |  | Annual | e | Middle | Arable | Europe | Dfb |
| Chirinda et al. 2010 | 0.201 | 0.003 |  |  |  |  | 1.95 |  | ORG only | ORG and CON |  | Similar |  | Annual | e | Middle | Arable | Europe | Dfb |
| Chirinda et al. 2010 | 0.260 | 0.016 |  |  |  |  | 2.15 |  | ORG only | ORG and CON |  | Different |  | Annual | e | Middle | Arable | Europe | Dfb |
| Gajda and Martyniuk, 2004 | 0.128 | 0.042 |  |  | 6.6 |  | 0.78 |  | ORG only | ORG only | CON only | Different |  | Annual | e | Middle | Arable | Europe | Dfb |
| Gajda and Martyniuk, 2004 | 0.405 | 0.049 |  |  | 6.5 |  | 0.82 |  | ORG only | ORG only | CON only | Different |  | Annual | e | Middle | Arable | Europe | Dfb |
| Ge et al. 2011 | 1.629 | 0.003 | 1255 | 17.5 | 8.2 | 20.0 | 1.21 | 0.155 | ORG only | ORG only | CON only | Different | Middle | Annual | e |  | Arable | Asia | Cfa |
| Ge et al. 2011 | 0.762 | 0.000 | 1255 | 17.5 | 7.8 | 20.0 | 1.75 | 0.252 | ORG only | ORG only | CON only | Different | Middle | Annual | e |  | Arable | Asia | Cfa |
| Ge et al. 2013 | 0.522 | 0.025 | 1255 | 17.5 | 7.9 | 21.0 | 1.52 | 0.222 | ORG only | none | CON only | Different | Middle | Annual | e | Middle | Arable | Asia | Cfa |
| Ge et al. 2013 | 1.841 | 0.099 | 1255 | 17.5 | 8.3 | 21.0 | 1.11 | 0.143 | ORG only | none | CON only | Different | Middle | Annual | e | Middle | Arable | Asia | Cfa |
| Glover et al. 2000 | 0.000 | 0.029 |  |  | 6.4 |  | 1.53 | 0.169 | ORG and CON | none | CON only |  |  | Perennial | e | Middle | Orchard | North America | BWk |
| Glover et al. 2000 | 0.247 | 0.039 |  |  | 6.5 |  | 1.40 | 0.156 | ORG only | none | CON only |  |  | Perennial | e | Middle | Orchard | North America | BWk |
| Gonzales-Perez et al. 2015 | 0.851 | 0.046 | 1255 | 17.5 | 7.0 | 21.0 | 2.27 | 0.280 | ORG only | ORG and CON | CON only |  | Middle | Annual | e | Old | Arable | Asia | Cfa |
| Gunapala et al. 1998 | 1.089 | 0.085 | 635 |  |  |  |  |  | ORG only | ORG only | CON only | Different |  | Annual | e | Middle | Arable | North America | Csa |
| Heinze et al. 2010 | 0.105 | 0.032 | 590 | 9.5 | 6.6 | 5.0 | 0.85 | 0.079 | ORG only | ORG and CON |  | Similar | Light | Annual | e | Old | Arable | Europe | Cfb |
| Heinze et al. 2010 | 0.154 | 0.032 | 590 | 9.5 | 6.6 | 5.0 | 0.86 | 0.080 | ORG only | ORG and CON |  | Similar | Light | Annual | e | Old | Arable | North America | Cfb |
| Kramer et al. 2006 | 0.430 | 0.037 |  |  |  | 6.4 | 1.89 | 0.186 | ORG and CON | none | CON only | Similar | Light | Perennial | f | Middle | Orchard | North America | BWk |
| Kramer et al. 2006 | 0.585 | 0.043 |  |  |  | 6.5 | 1.64 | 0.160 | ORG only | none | CON only | Similar | Light | Perennial | e | Middle | Orchard | North America | BWk |
| Larsen et al. 2014 | 2.047 | 1.381 | 1084 | 12.6 |  |  | 1.26 |  |  | ORG only | CON only |  |  | Annual | f | Old | Arable | North America | Cfb |
| Larsen et al. 2014 | 1.452 | 0.461 | 1084 | 12.6 |  |  | 1.06 |  |  | ORG only | CON only |  |  | Annual | f | Old | Arable | North America | Cfb |
| Larsen et al. 2014 | 1.001 | 1.337 | 1084 | 12.6 |  |  | 1.01 |  |  | ORG only | CON only |  |  | Annual | f | Old | Arable | North America | Cfb |
| Larsen et al. 2014 | 1.001 | 1.337 | 1084 | 12.6 |  |  | 0.81 |  |  | ORG only | CON only |  |  | Annual | f | Old | Arable | North America | Cfb |
| Liebig and Doran 1999 | 0.629 | 0.060 |  |  | 6.1 |  |  |  | ORG only | ORG only |  | Different |  | Annual | f | Middle | Arable | North America | Dfa |
| Liebig and Doran 1999 | 1.264 | 0.152 |  |  | 5.7 |  |  |  | none | ORG and CON |  | Different |  | Annual | f | Old | Arable | North America | Dfa |
| Liebig and Doran 1999 | 0.804 | 0.075 |  |  | 6.4 |  |  |  | ORG only | ORG and CON |  | Different |  | Annual | f | Middle | Arable | North America | Dfa |
| Liebig and Doran 1999 | 0.665 | 0.063 |  |  | 7.4 |  |  |  | ORG only | ORG only |  | Different |  | Annual | f | Old | Arable | North America | Dfa |
| Liebig and Doran 1999 | -0.044 | 0.042 |  |  | 6.8 |  |  |  | ORG only | ORG only |  | Different |  | Annual | f | Old | Arable | North America | Dfa |
| Liu et al. 2007 | 0.646 | 0.062 |  |  |  |  | 1.63 | 0.277 | ORG only | ORG only | CON only | Different |  | Annual | f | Old | Arable | North America | Cfa |
| Liu et al. 2007 | 1.339 | 0.110 |  |  |  |  | 1.18 | 0.232 | ORG only | ORG only | CON only | Different |  | Annual | f | Old | Arable | North America | Cfa |
| Marinari et al. 2006 | 0.324 | 0.023 |  |  | 7.6 | 20.0 | 0.96 | 0.100 | ORG only | none | CON only | Similar | Middle | Annual | f | Middle | Arable | Europe | Csa |
| Marinari et al. 2010 | 0.289 | 0.060 |  |  |  |  | 1.19 | 0.135 | ORG only | ORG only | CON only | Similar |  | Annual | e | Middle | Arable | Europe | Csa |
| Melero et al. 2006 | 1.040 | 0.116 |  |  | 8.4 | 23.0 | 1.52 | 0.175 | ORG only | ORG and CON | none | Similar | Middle | Annual | e | Middle | Arable | Europe | Csa |
| Melero et al. 2006 | 0.598 | 0.049 |  |  | 7.8 | 23.0 | 1.46 | 0.155 | ORG only | ORG and CON | none | Similar | Middle | Annual | e | Middle | Orchard | Europe | Csa |
| Monokrousos et al. 2006 | -0.427 | 0.008 | 506 |  | 7.7 | 26.1 | 1.43 | 0.067 | ORG and CON | none | CON only | Similar | Middle | Annual | f | Middle | Arable | Europe | Cfa |
| Monokrousos et al. 2006 | -0.302 | 0.008 | 506 |  | 7.9 | 29.6 | 1.61 | 0.073 | ORG and CON | none | CON only | Similar | Middle | Annual | f | Middle | Arable | Europe | Cfa |
| Monokrousos et al. 2006 | -0.363 | 0.010 | 506 |  | 7.9 | 28.2 | 1.42 | 0.068 | ORG and CON | none | CON only | Similar | Middle | Annual | f | Young | Arable | Europe | Cfa |
| Murata et al. 1997 | 0.336 | 0.041 | 1050 |  |  |  | 3.43 | 0.336 | ORG and CON | ORG and CON |  | Different |  | Annual | f | Middle | Arable | Oceania | Cfb |
| Murata et al. 1997 | 0.122 | 0.035 | 1050 |  |  |  | 3.44 | 0.337 | ORG and CON | ORG and CON |  | Different |  | Perennial | f | Middle | Grassland | Oceania | Cfb |
| Basalrespiration |  |  |  |  |  |  |  |  |  |  |  |  |  |  |  |  |  |  |  |
| Amaral et al. 2012 | -0.320 | 0.016 |  |  | 5.3 | 20.6 | 0.53 |  | ORG only | ORG only | CON only | Different | Middle | Perennial | e |  | Orchard | South America | Cfa |
| Amaral et al. 2012 | -1.363 | 0.011 |  |  | 5.3 | 20.6 | 0.53 |  | ORG only | ORG only | CON only | Different | Middle | Perennial | e |  | Orchard | South America | Cfa |
| Anonymous, Unpublsihed | 0.102 | 0.029 | 1950 | 20.0 | 6.1 |  | 2.55 | 0.181 | ORG and CON | ORG and CON | CON only | Different |  | Annual | e | Middle | Arable | Africa | Aw |
| Anonymous, Unpublsihed | 0.288 | 0.032 | 1000 | 20.0 | 6.4 |  | 2.14 | 0.248 | ORG and CON | ORG and CON | CON only | Different |  | Annual | e | Middle | Arable | Africa | Cfb |
| Bending et al. 2004 | -0.070 | 0.029 |  |  |  | 14.0 | 1.19 | 0.089 | ORG only | ORG only |  | Different | Light | Annual | e | Middle | Arable | Europe | Cfb |
| Bending et al. 2004 | -0.233 | 0.025 |  |  |  | 14.0 | 1.12 | 0.084 | ORG only | ORG only |  | Different | Light | Annual | e | Middle | Arable | Europe | Cfb |
| Bending et al. 2004 | -0.016 | 0.031 |  |  |  | 14.0 | 1.07 | 0.079 | ORG only | ORG only |  | Different | Light | Annual | e | Middle | Arable | Europe | Cfb |
| Bending et al. 2004 | -0.088 | 0.029 |  |  |  | 14.0 | 1.11 | 0.084 | ORG only | ORG only |  | Different | Light | Annual | e | Middle | Arable | Europe | Cfb |
| Bossio et al. 1998 | 0.118 | 0.032 | 635 |  |  |  |  |  | ORG only | ORG only | CON only | Different |  | Annual | e | Middle | Arable | North America | Csb |
| Bossio et al. 1998 | -0.105 | 0.032 | 635 |  |  |  |  |  | none | ORG and CON | CON only | Different |  | Annual | e | Middle | Arable | North America | Csb |
| Burger et al. 2005 | -0.094 | 0.018 | 499 | 16.2 | 6.7 | 22.5 | 1.27 | 0.120 | ORG only | ORG only | . | Different | Middle | Annual | e | Old | Arable | North America | Csa |
| Carey et al. 2009 | 0.114 | 0.004 | 1735 | 14.7 | 6.6 |  | 5.40 | 0.450 |  | ORG and CON |  |  |  | Annual | fa | Middle | Arable | Oceania | Cfb |
| Carey et al. 2009 | 0.124 | 0.004 | 1735 | 14.7 | 6.6 |  | 5.65 | 0.470 |  | ORG and CON |  |  |  | Annual | f | Middle | Arable | Oceania | Cfb |
| Carpenter-Boggs et al. 2000 | 1.099 | 0.156 | 550 |  |  |  | 2.03 |  | ORG only | ORG and CON |  | Similar | Light | Annual | e |  | Arable | North America | BSk |
| Carpenter-Boggs et al. 2000 | 1.014 | 0.134 | 550 |  |  |  | 2.03 |  | ORG only | ORG and CON |  | Similar |  | Annual | e |  | Arable | North America | BSk |
| Chirinda et al. 2010 | 0.235 | 0.066 |  |  |  |  | 2.10 |  | none | ORG and CON |  | Similar |  | Annual | ee | Middle | Arable | Europe | Dfb |
| Chirinda et al. 2010 | 0.336 | 0.070 |  |  |  |  | 1.95 |  | ORG only | ORG and CON |  | Similar |  | Annual | e | Middle | Arable | Europe | Dfb |
| Chirinda et al. 2010 | 0.496 | 0.079 |  |  |  |  | 2.15 |  | ORG only | ORG and CON |  | Similar |  | Annual | e | Middle | Arable | Europe | Dfb |
| Chirinda et al. 2010 | 0.299 | 0.068 |  |  |  |  | 2.15 |  | ORG only | ORG and CON |  | Different |  | Annual | e | Middle | Arable | Europe | Dfb |
| Fliessbach et al. 2007 | -0.006 | 0.010 | 785 | 9.5 | 6.3 | 15.0 | 1.32 | 0.143 | ORG only | ORG and CON | CON only | Similar | Light | Annual | e | Old | Arable | Europe | Cfb |
| Fliessbach et al. 2007 | 0.035 | 0.011 | 785 | 9.5 | 6.5 | 15.0 | 1.33 | 0.142 | ORG and CON | ORG and CON | CON only | Similar | Light | Annual | e | Old | Arable | Europe | Cfb |
| Fliessbach et al. 2007 | 0.081 | 0.011 | 785 | 9.5 | 6.5 | 15.0 | 1.40 | 0.153 | ORG only | ORG and CON | CON only | Similar | Light | Annual | e | Old | Arable | Europe | Cfb |
| Fliessbach et al. 2007 | 0.123 | 0.012 | 785 | 9.5 | 6.7 | 15.0 | 1.41 | 0.152 | ORG and CON | ORG and CON | CON only | Similar | Light | Annual | e | Old | Arable | Europe | Cfb |
| Fraser et al. 1989 | 0.387 | 0.050 | 680 | 11.0 | 7.0 |  | 1.88 |  | ORG only | ORG and CON | CON only | Similar |  | Annual | e | Middle | Arable | North America | Cfa |
| Freitas et al. 2011 | 1.100 | 0.063 | 450 | 26.0 | 7.0 | 30.0 | 0.91 |  | ORG only | none |  | Similar | Middle | Perennial | f | Young | Orchard | South America | BSh |
| Gajda and Martyniuk, 2004 | 0.278 | 0.057 |  |  | 6.6 |  | 0.78 |  | ORG only | ORG only | CON only | Different |  | Annual | e | Middle | Arable | Europe | Dfb |
| Gajda and Martyniuk, 2004 | 0.376 | 0.065 |  |  | 6.5 |  | 0.82 |  | ORG only | ORG only | CON only | Different |  | Annual | e | Middle | Arable | Europe | Dfb |
| Ge et al. 2011 | 2.042 | 0.640 | 1255 | 17.5 | 8.2 | 20.0 | 1.21 | 0.155 | ORG only | none | CON only | Different | Middle | Annual | e |  | Arable | Asia | Cfa |
| Ge et al. 2011 | 3.949 | 28.083 | 1255 | 17.5 | 7.8 | 20.0 | 1.75 | 0.252 | ORG only | ORG only | CON only | Different | Middle | Annual | e |  | Arable | Asia | Cfa |
| Heinze et al. 2010 | -0.058 | 0.030 | 590 | 9.5 | 6.6 | 5.0 | 0.85 | 0.079 | ORG only | ORG and CON |  | Similar |  | Annual | e | Old | Arable | Europe | Cfb |
| Heinze et al. 2010 | 0.019 | 0.032 | 590 | 9.5 | 6.6 | 5.0 | 0.86 | 0.080 | ORG only | ORG and CON |  | Similar | Light | Annual | e | Old | Arable | Europe | Cfb |
| Islam and Weil 2000 | 0.226 | 0.040 |  |  | 6.1 | 27.0 | 2.35 | 0.313 | ORG only | ORG and CON |  | Different | Middle | Annual | e | Old | Arable | North America | Dfa |
| Islam and Weil 2000 | 0.180 | 0.038 |  |  | 6.1 | 27.1 | 2.39 | 0.308 | none | ORG and CON |  | Different | Middle | Annual | e | Old | Arable | North America | Dfa |
| Jacinthe et al. 2011 | -1.815 | 2.088 | 205 | 20.0 | 7.8 |  | 0.90 |  | ORG only | ORG only |  | Different |  | Annual | f | Young | Arable | South America | BSh |
| Jacinthe et al. 2011 | -1.400 | 1.134 | 205 | 20.0 | 7.7 |  | 0.84 |  | ORG only | ORG only |  | Different |  | Annual | f | Middle | Arable | South America | BSh |
| Jacinthe et al. 2011 | -0.575 | 0.356 | 205 | 20.0 | 7.8 |  | 0.75 |  | ORG only | ORG only |  | Different |  | Annual | f | Middle | Arable | South America | BSh |
| Lagomarsino et al. 2009 | 0.175 | 0.043 |  |  |  |  | 2.10 |  | none | ORG and CON |  | Different |  | Annual | e | Middle | Arable | Europe | Csa |
| Lagomarsino et al. 2009 | 0.072 | 0.042 |  |  |  |  | 1.95 |  | ORG only | ORG and CON |  | Different |  | Annual | e | Middle | Arable | Europe | Csa |
| Lagomarsino et al. 2009 | 0.022 | 0.042 |  |  |  |  | 2.15 |  | ORG only | ORG and CON |  | Different |  | Annual | e | Middle | Arable | Europe | Csa |
| Liu et al. 2007 | 1.270 | 0.285 |  |  |  |  | 1.63 | 0.277 | ORG only | ORG only | CON only | Different |  | Annual | f | Old | Arable | North America | Cfa |
| Liu et al. 2007 | 2.409 | 2.600 |  |  |  |  | 1.18 | 0.232 | ORG only | ORG only | CON only | Different |  | Annual | f | Old | Arable | North America | Cfa |
| Marinari et al. 2010 | 0.366 | 0.024 | 954 | 14.2 | 7.4 |  | 2.82 | 0.165 | ORG only | ORG only |  | Different |  | Annual | f | Old | Arable | Europe | Csa |
| Marinari et al. 2010 | 0.278 | 0.034 | 792 | 9.5 | 5.4 |  | 1.41 | 0.175 | ORG only | ORG and CON |  | Similar |  | Annual | e | Old | Arable | Europe | Cfb |
| Marinari et al. 2010 | 0.641 | 0.031 | 420 | 12.9 | 6.0 |  | 0.95 | 0.100 | ORG and CON | ORG only |  | Different |  | Annual | e | Old | Arable | Europe | Csa |
| Marinari et al. 2010 B | 0.304 | 0.015 |  |  |  |  | 1.19 | 0.135 | ORG only | ORG only | CON only | Similar |  | Annual | e | Middle | Arable | Europe | Csa |
| Mazzoncini et al. 2010 | 0.165 | 0.050 | 700 |  |  |  | 0.87 | 0.105 | ORG only | ORG and CON |  | Similar |  | Annual | e | Middle | Arable | Europe | Csa |
| Melero et al. 2006 | 0.562 | 0.015 |  |  | 8.4 | 23.0 | 1.52 | 0.175 | ORG only | ORG and CON | none | Similar | Middle | Annual | e | Middle | Arable | Europe | Csa |
| Melero et al. 2006 | 0.183 | 0.021 |  |  | 7.8 | 23.0 | 1.46 | 0.155 | ORG only | ORG and CON | none | Similar | Middle | Annual | e | Middle | Orchard | Europe | Csa |
| Monokrousos et al. 2006 | 0.037 | 0.025 | 506 |  | 7.7 | 26.1 | 1.43 | 0.067 | ORG and CON | none | CON only | Similar | Middle | Annual | f | Middle | Arable | Europe | Cfa |
| Monokrousos et al. 2006 | -0.230 | 0.026 | 506 |  | 7.9 | 29.6 | 1.61 | 0.073 | ORG and CON | none | CON only | Similar | Middle | Annual | f | Middle | Arable | Europe | Cfa |
| Monokrousos et al. 2006 | 0.097 | 0.025 | 506 |  | 7.9 | 28.2 | 1.42 | 0.068 | ORG and CON | none | CON only | Similar | Middle | Annual | f | Young | Arable | Europe | Cfa |
| Reganold et al. 1993 | 1.466 | 0.112 |  |  | 6.8 |  | 3.68 | 0.460 | ORG only |  | CON only |  |  | Annual | f | Old | Arable | Oceania | Cfb |
| Reganold et al. 1993 | 0.232 | 0.015 |  |  | 6.6 |  | 4.32 | 0.460 | ORG only |  | CON only |  |  | Perennial | f | Old | Orchard | Oceania | Cfb |
| Reganold et al. 1993 | 0.474 | 0.020 |  |  | 6.3 |  | 6.03 | 0.460 | ORG only |  | CON only |  |  | Perennial | f | Middle | Orchard | Oceania | Cfb |
| Reganold et al. 1993 | 0.269 | 0.015 |  |  | 5.7 |  | 4.94 | 0.460 | ORG only |  | CON only |  |  | Annual | f | Middle | Arable | Oceania | Cfb |
| Reganold et al. 1993 | 0.016 | 0.012 |  |  | 6.2 |  | 3.41 | 0.460 | ORG only |  | CON only |  |  |  | f | Old |  | Oceania | Cfb |
| Reganold et al. 1993 | -0.053 | 0.011 |  |  | 5.7 |  | 5.05 | 0.460 | ORG only |  | CON only |  |  | Perennial | f | Old | Grassland | Oceania | Cfb |
| Reganold et al. 1993 | 0.313 | 0.016 |  |  | 6.1 |  | 3.79 | 0.460 | ORG only |  | CON only |  |  | Perennial | f | Middle | Grassland | Oceania | Cfb |
| Reganold et al. 2010 | 0.288 | 0.010 |  |  | 7.1 | 13.1 | 0.91 | 0.077 | ORG and CON | ORG and CON | CON only |  | Light | Perennial | f |  | Orchard | North America | Csb |
| Sudhakaran et al 2013 | 0.572 | 0.057 | 1242 |  | 7.2 |  | 0.75 | 0.295 | ORG and CON | ORG and CON | CON only |  |  | Annual | f |  | Arable | Asia | Aw |
| Sudhakaran et al 2013 | 0.516 | 0.054 | 1242 |  | 7.4 |  | 0.79 | 0.275 | ORG and CON | ORG and CON | none |  |  | Annual | f |  | Arable | Asia | Aw |
| Van Diepeningen et al. 2006 | 0.527 | 0.087 |  |  | 7.4 | 11.7 | 2.03 | 0.161 | ORG only |  |  |  | Light | Annual | f |  | Arable | Europe | Cwc |
| Van Diepeningen et al. 2006 | 0.000 | 0.089 |  |  | 5.4 | 4.0 | 2.69 | 0.164 | ORG only |  |  |  | Light | Annual | f |  | Arable | Europe | Cwc |
| Velmourougane 2016 | 0.167 | 0.030 | 1700 |  | 5.4 | 25.0 | 2.00 | 0.315 | ORG only | none | CON only | Different | Middle | Perennial | e | Old | Orchard | Asia | Am |
| Wander et al. 1995 | 0.445 | 0.027 |  |  |  |  |  |  | ORG only | ORG only | CON only | Different |  | Annual | e | Middle | Arable | North America | Dfa |
| Wander et al. 1995 | 0.155 | 0.018 |  |  |  |  |  |  | ORG only | ORG only | CON only | Different |  | Annual | e | Middle | Arable | North America | Dfa |
| Total PLFA |  |  |  |  |  |  |  |  |  |  |  |  |  |  |  |  |  |  |  |
| Burger et al. 2005 | 0.570 | 0.007 | 499 | 16.2 | 6.7 | 22.5 | 1.27 | 0.120 | ORG only | ORG only | . | Different | Middle | Annual | e | Old | Arable | North America | Csa |
| Esperschütz et al. 2007 | 0.212 | 0.021 | 785 | 9.5 | 6.3 | 15.0 | 1.36 | 0.161 | ORG only | ORG and CON | CON only | Similar | Light | Annual | e | Old | Arable | Europe | Cfb |
| Esperschütz et al. 2007 | 0.371 | 0.023 | 785 | 9.5 | 6.5 | 15.0 | 1.37 | 0.159 | ORG and CON | ORG and CON | CON only | Similar | Light | Annual | e | Old | Arable | Europe | Cfb |
| Esperschütz et al. 2007 | 0.271 | 0.022 | 785 | 9.5 | 6.5 | 15.0 | 1.34 | 0.175 | ORG only | ORG and CON | CON only | Similar | Light | Annual | e | Old | Arable | Europe | Cfb |
| Esperschütz et al. 2007 | 0.131 | 0.020 | 785 | 9.5 | 6.7 | 15.0 | 1.50 | 0.173 | ORG and CON | ORG and CON | CON only | Similar | Light | Annual | e | Old | Arable | Europe | Cfb |
| Ge et al. 2013 | 0.979 | 0.003 | 1255 | 17.5 | 7.9 | 21.0 | 1.52 | 0.222 | ORG only | none | CON only | Different | Middle | Annual | e | Middle | Arable | Asia | Cfa |
| Ge et al. 2013 | 1.060 | 0.003 | 1255 | 17.5 | 8.3 | 21.0 | 1.11 | 0.143 | ORG only | none | CON only | Different | Middle | Annual | e | ; | Arable | Asia | Cfa |
| Kong et al. 2011 | 0.335 | 0.030 | 557 | 17.6 |  |  | 1.30 | 0.159 | ORG and CON | ORG and CON | . | Different |  | Annual | e | Old | Arable | Nort America | Csa |
| Kong et al. 2011 | 0.291 | 0.029 | 557 | 17.6 |  |  | 1.33 | 0.163 | ORG only | ORG only | . | Different |  | Annual | e | Old | Arable | Nort America | Csa |
| Moeskops et al. 2010 | 0.506 | 0.001 | 3035 | 21.3 | 4.5 |  | 3.07 | 0.330 | ORG and CON | none | CON only | Similar |  | Annual | fa | Middle | Arable | Asia | Af |
| Moeskops et al. 2010 | 0.740 | 0.015 | 3035 | 21.3 | 4.5 |  | 3.05 | 0.350 | ORG and CON | none | CON only | Similar |  | Annual | f | Middle | Arable | Asia | Af |
| Moeskops et al. 2010 | 0.821 | 0.007 | 3035 | 21.3 | 5.0 |  | 4.80 | 0.505 | ORG and CON | none | CON only | Similar |  | Annual | f | Middle | Arable | Asia | Af |
| Moeskops et al. 2010 | 0.443 | 0.004 | 3035 | 21.3 | 5.6 |  | 5.05 | 0.530 | ORG and CON | none | CON only | Similar |  | Annual | f | Middle | Arable | Asia | Af |
| Moeskops et al. 2010 | 0.583 | 0.011 | 3035 | 21.3 | 4.8 |  | 3.59 | 0.410 | ORG and CON | none | CON only | Similar |  | Annual | f | Young | Arable | Asia | Af |
| Moeskops et al. 2010 | 0.625 | 0.005 | 3035 | 21.3 | 4.9 |  | 3.17 | 0.365 | ORG and CON | none | CON only | Similar |  | Annual | f | Young | Arable | Asia | Af |
| Romaniuk et al. 2011 | 0.163 | 0.014 | 1023 | 16.3 |  |  |  |  | ORG only | ORG and CON | CON only |  |  | Annual | f | Middle | Arable | South America | Cfa |
| Romaniuk et al. 2011 | 0.378 | 0.019 | 1023 | 16.3 |  |  |  |  | ORG only | ORG and CON | CON only |  |  | Annual | f | Old | Arable | South America | Cfa |
| Wander et al. 1995 | 0.092 | 0.137 |  |  |  |  |  |  | ORG only | ORG only | CON only | Different |  | Annual | e | Middle | Arable | Nort America | Dfa |
| Wander et al. 1995 | 0.238 | 0.221 |  |  |  |  |  |  | ORG only | ORG only | CON only | Different |  | Annual | e | Middle | Arable | Nort America | Dfa |
| Yeates et al. 1997 | 0.589 | 0.022 | 1230 | 9.5 | 5.6 | 49.0 | 5.10 |  | ORG only | ORG and CON |  | Similar | Heavy | Perennial | f |  | Grassland | Europe | Cfb |
| Yeates et al. 1997 | -0.094 | 0.016 | 1040 | 9.6 | 6.0 | 37.6 | 5.85 |  | none | ORG and CON |  | Similar | Middle | Perennial | f |  | Grassland | Europe | Cfb |
| Yeates et al. 1997 | 0.180 | 0.017 | 820 | 9.6 | 6.1 | 28.2 | 5.70 |  | only con | ORG and CON |  | Similar | Middle | Perennial | f |  | Grassland | Europe | Cfb |
| Dehydrogenase |  |  |  |  |  |  |  |  |  |  |  |  |  |  |  |  |  |  |  |
| Anonymous, Unpublsihed | 0.208 | 0.038 | 1950 | 20.0 | 6.1 |  | 2.55 | 0.181 | ORG and CON | ORG and CON | CON only | Different |  | Annual | Experimantal | Middle | Arable | Africa | Aw |
| Anonymous, Unpublsihed | 0.213 | 0.038 | 1000 | 20.0 | 6.4 |  | 2.14 | 0.248 | ORG and CON | ORG and CON | CON only | Different |  | Annual | Experimantal | Middle | Arable | Africa | Cfb |
| Aparna et al. 2014 1 | 0.876 | 0.014 |  |  | 8.3 | 17.0 | 0.55 |  | ORG only |  | CON only | Similar | Middle | Perennial | Farm | | Orchard | Asia | BWh |
| Aparna et al. 2014 2 | 1.243 | 0.017 |  |  | 8.3 | 17.0 | 0.55 |  | ORG only |  | CON only | Similar | Middle | Perennial | Farm | | Orchard | Asia | BWh |
| Benitez et al. 2006 | 0.449 | 0.224 |  |  | 7.2 |  | 1.14 |  | ORG and CON | none | CON only | Similar |  | Perennial | Farm | Young | Orchard | Europe | BSk |
| Benitez et al. 2006 | 0.460 | 0.258 |  |  | 7.3 |  | 1.08 |  | ORG only | none | CON only | Similar |  | Perennial | Farm | Young | Orchard | Europe | BSk |
| Bolton Jr et al. 1985 | 0.529 | 0.029 |  |  | 5.8 | . | 1.03 | 0.112 | ORG only | ORG and CON | . | Different |  | Annual | Farm | Old | Arable | North America | BSk |
| Carpenter-Boggs et al. 2000 1 | 0.615 | 0.064 | 550 |  |  |  | 2.03 |  | ORG only | ORG and CON |  | Similar |  | Annual | Experimantal | | Arable | North America | BSk |
| Carpenter-Boggs et al. 2000 2 | 0.539 | 0.059 | 550 |  |  |  | 2.03 |  | ORG only | ORG and CON |  | Similar |  | Annual | Experimantal | | Arable | North America | BSk |
| Fliessbach et al. 2007 1 | 0.699 | 0.024 | 785 | 9.5 | 6.3 | 15.0 | 1.32 | 0.143 | ORG only | ORG and CON | CON only | Similar | Light | Annual | Experimantal | Old | Arable | Europe | Cfb |
| Fliessbach et al. 2007 2 | 0.282 | 0.016 | 785 | 9.5 | 6.5 | 15.0 | 1.33 | 0.142 | ORG and CON | ORG and CON | CON only | Similar | Light | Annual | Experimantal | Old | Arable | Europe | Cfb |
| Fliessbach et al. 2007 3 | 0.955 | 0.033 | 785 | 9.5 | 6.5 | 15.0 | 1.40 | 0.153 | ORG only | ORG and CON | CON only | Similar | Light | Annual | Experimantal | Old | Arable | Europe | Cfb |
| Fliessbach et al. 2007 4 | 0.538 | 0.020 | 785 | 9.5 | 6.7 | 15.0 | 1.41 | 0.152 | ORG and CON | ORG and CON | CON only | Similar | Light | Annual | Experimantal | Old | Arable | Europe | Cfb |
| Gajda and Martyniuk, 2004 1 | 0.230 | 0.063 |  |  | 6.6 |  | 0.78 |  | ORG only | ORG only | CON only | Different |  | Annual | Experimantal | Middle | Arable | Europe | Dfb |
| Gajda and Martyniuk, 2004 2 | 0.567 | 0.081 |  |  | 6.5 |  | 0.82 |  | ORG only | ORG only | CON only | Different |  | Annual | Experimantal | Middle | Arable | Europe | Dfb |
| Garcia-Ruiz et al 2009 1 | 0.134 | 0.298 | 560 | 16.0 | 7.9 | 28.8 | 8.05 | 0.295 | ORG only |  | CON only |  | Middle | Perennial | f | Middle | Orchard | Europe | BSk |
| Garcia-Ruiz et al 2009 2 | 0.558 | 0.235 | 560 | 16.0 | 7.6 | 41.4 | 5.10 | 0.185 | ORG only |  | CON only |  | Heavy | Perennial | f | Middle | Orchard | Europe | BSk |
| Garcia-Ruiz et al 2009 3 | 0.905 | 0.293 | 560 | 16.0 | 7.7 | 21.2 | 8.35 | 0.170 | ORG only |  | CON only |  | Middle | Perennial | f | Middle | Orchard | Europe | BSk |
| Järvan et al. 2014 1 | -0.156 | 0.061 | 726 | 5.3 | 6.1 | 38.4 | 1.55 |  | ORG only | ORG and CON | CON only | Different | Middle | Annual | Experimantal | | Arable | Europe | Dfb |
| Järvan et al. 2014 2 | 0.244 | 0.064 | 726 | 5.3 | 6.1 | 38.4 | 1.55 |  | ORG and CON | ORG and CON | CON only | Different | Middle | Annual | Experimantal | | Arable | Europe | Dfb |
| Lagomarsino et al. 2009 1 | 0.015 | 0.252 | 954 | 14.2 | 6.8 |  | 1.21 | 0.150 | ORG only | ORG only | CON only | Different |  | Annual | Experimantal | Middle | Arable | Europe | Csa |
| Lagomarsino et al. 2009 2 | -0.538 | 2.204 | 954 | 14.2 | 6.9 |  | 1.24 | 0.130 | ORG only | ORG only | CON only | Different |  | Annual | Experimantal | Middle | Arable | Europe | Csa |
| Lagomarsino et al. 2009 3 | 0.524 | 0.264 | 954 | 14.2 | 6.9 |  | 1.24 | 0.155 | ORG only | ORG only | CON only | Different |  | Annual | Experimantal | Middle | Arable | Europe | Csa |
| Marinari et al. 2006 | 1.366 | 0.131 |  |  | 7.6 | 20.0 | 0.96 | 0.100 | ORG only | none | CON only | Similar | Middle | Annual | f | Middle | Arable | Europe | Csa |
| Moeskops et al. 2010 | 1.476 | 0.683 | 3035 | 21.3 | 4.5 |  | 3.07 | 0.330 | ORG and CON | none | CON only | Similar |  | Annual | f | Middle | Arable | Asia | Af |
| Moeskops et al. 2010 | 1.714 | 0.144 | 3035 | 21.3 | 4.5 |  | 3.05 | 0.350 | ORG and CON | none | CON only | Similar |  | Annual | f | Middle | Arable | Asia | Af |
| Moeskops et al. 2010 | 1.816 | 0.435 | 3035 | 21.3 | 5.0 |  | 4.80 | 0.505 | ORG and CON | none | CON only | Similar |  | Annual | f | Middle | Arable | Asia | Af |
| Moeskops et al. 2010 | 1.514 | 1.438 | 3035 | 21.3 | 5.6 |  | 5.05 | 0.530 | ORG and CON | none | CON only | Similar |  | Annual | f | Middle | Arable | Asia | Af |
| Moeskops et al. 2010 | 1.953 | 0.176 | 3035 | 21.3 | 4.8 |  | 3.59 | 0.410 | ORG and CON | none | CON only | Similar |  | Annual | f | Young | Arable | Asia | Af |
| Moeskops et al. 2010 | 1.392 | 0.465 | 3035 | 21.3 | 4.9 |  | 3.17 | 0.365 | ORG and CON | none | CON only | Similar |  | Annual | f | Young | Arable | Asia | Af |
| Okur et al. 2009 1 | 0.615 | 0.008 | 706 | 17.0 | 7.5 | 15.9 | 0.77 | 0.135 | ORG only | none | CON only | Similar | Light | Perennial | f |  | Orchard | Asia | Csa |
| Okur et al. 2009 2 | 0.518 | 0.008 | 706 | 17.0 | 7.5 | 15.9 | 0.77 | 0.135 | ORG only | none | CON only | Similar | Light | Perennial | f |  | Orchard | Asia | Csa |
| Okur et al. 2009 3 | 0.276 | 0.013 | 706 | 17.0 | 7.5 | 15.9 | 0.77 | 0.135 | ORG only | none | CON only | Similar | Light | Perennial | f |  | Orchard | Asia | Csa |
| Reganold et al. 2010 | 0.753 | 0.064 |  |  | 7.1 | 13.1 | 0.91 | 0.077 | ORG and CON | ORG and CON | no |  | Light | Perennial | f | Middle | Orchard | North America | Csb |
| Stark et al. 2008 | 0.190 | 0.463 | 680 | 11.5 | 5.9 |  | 2.35 | 0.230 | ORG and CON | ORG and CON |  | Different |  | Annual | Experimantal | | Arable | Oceania | Cfb |
| Stark et al. 2008 | 0.477 | 0.143 | 680 | 11.5 | 5.9 |  | 2.35 | 0.230 | ORG and CON | ORG and CON |  | Different |  | Annual | Experimantal | | Arable | Oceania | Cfb |
| Velmourougane 2016 | 0.177 | 0.037 | 1700 |  | 5.4 | 25.0 | 2.00 | 0.315 | ORG only | none | CON only | Different | Middle | Perennial | Experimantal | Old | Orchard | Asia | Am |
| Yeates et al. 1997 1 | 0.431 | 0.022 | 1230 | 9.5 | 5.6 | 49.0 | 5.10 |  | ORG only | ORG and CON |  | Similar | Heavy | Perennial | f |  | Grassland | Europe | Cfb |
| Yeates et al. 1997 2 | -0.176 | 0.019 | 1040 | 9.6 | 6.0 | 37.6 | 5.85 |  | none | ORG and CON |  | Similar | Middle | Perennial | f |  | Grassland | Europe | Cfb |
| Yeates et al. 1997 3 | 0.336 | 0.020 | 820 | 9.6 | 6.1 | 28.2 | 5.70 |  | CON only | ORG and CON |  | Similar | Middle | Perennial | f |  | Grassland | Europe | Cfb |
| Metabolic quotient |  |  |  |  |  |  |  |  |  |  |  |  |  |  |  |  |  |  |  |
| Amaral et al. 2012 | -1.420 | 0.064 |  |  | 5.3 | 20.6 | 0.53 |  | ORG only | ORG only | CON only | Different | Middle | Perennial | Experimental | Middle | Orchard | Southamerica | Cfa |
| Amaral et al. 2012 | -2.643 | 0.665 |  |  | 5.3 | 20.6 | 0.53 |  | ORG only | ORG only | CON only | Different | Middle | Perennial | Experimental | Middle | Orchard | Southamerica | Cfa |
| Anonymous, Unpublsihed | -0.368 | 0.018 | 1950 | 20.0 | 6.1 |  | 2.55 | 0.181 | ORG and CON | ORG and CON | CON only | Different |  | Annual | Experimental | Middle | Arable | Africa | Aw |
| Anonymous, Unpublsihed | 0.208 | 0.017 | 1000 | 20.0 | 6.4 |  | 2.14 | 0.248 | ORG and CON | ORG and CON | CON only | Different |  | Annual | Experimental | Middle | Arable | Africa | Cfb |
| Bossio et al. 1998 | 0.000 | 0.020 | 635 |  |  |  |  |  | ORG only | ORG only | CON only | Different |  | Annual | Experimental | Middle | Arable | North America | Csb |
| Bossio et al. 1998 | 0.000 | 0.020 | 635 |  |  |  |  |  | none | ORG and CON | CON only | Different |  | Annual | Experimental | Middle | Arable | North America | Csb |
| Burger et al. 2005 | -0.631 | 0.017 | 499 | 16.2 | 6.7 | 22.5 | 1.27 | 0.120 | ORG only | ORG only | . | Different | Middle | Annual | Experimental | Old | Arable | North America | Csa |
| Carey et al. 2009 | -0.068 | 0.002 | 1735 | 14.7 | 6.6 |  | 5.40 | 0.450 |  | ORG and CON |  |  |  | Annual | Farm | Middle | Arable | Oceania | Cfb |
| Carey et al. 2009 | 0.018 | 0.002 | 1735 | 14.7 | 6.6 |  | 5.65 | 0.470 |  | ORG and CON |  |  |  | Annual | Farm | Middle | Arable | Oceania | Cfb |
| Carpenter-Boggs et al. 2000 | 0.693 | 0.031 | 550 |  |  |  | 2.03 |  | ORG only | ORG and CON |  | Similar | Light | Annual | Experimental | Middle | Arable | North America | BSk |
| Carpenter-Boggs et al. 2000 | 0.649 | 0.030 | 550 |  |  |  | 2.03 |  | ORG only | ORG and CON |  | Similar |  | Annual | Experimental | Middle | Arable | North America | BSk |
| Fraser et al. 1989 | 0.387 | 0.023 | 680 | 11.0 | 7.0 |  | 1.88 |  | ORG only | ORG and CON | CON only | Similar |  | Annual | Experimental | Middle | Arable | North America | Dfa |
| Freitas et al. 2011 | 0.193 | 0.008 | 450 | 26.0 | 7.0 | 30.0 | 0.91 |  | ORG only | none |  | Similar | Middle | Perennial | Farm | Young | Orchard | South America | BSh |
| Gajda and Martyniuk, 2004 | 0.081 | 0.027 |  |  | 6.6 |  | 0.78 |  | ORG only | ORG only | CON only | Different |  | Annual | Experimental | Middle | Arable | Europe | Dfb |
| Gajda and Martyniuk, 2004 | -0.065 | 0.027 |  |  | 6.5 |  | 0.82 |  | ORG only | ORG only | CON only | Different |  | Annual | Experimental | Middle | Arable | Europe | Dfb |
| Ge et al. 2011 | 0.696 | 0.042 | 1255 | 17.5 | 8.2 | 20.0 | 1.21 | 0.155 | ORG only | none | CON only | Different | Middle | Annual | Experimental | | Arable | Asia | Cfa |
| Ge et al. 2011 | 2.329 | 0.716 | 1255 | 17.5 | 7.8 | 20.0 | 1.75 | 0.252 | ORG only | ORG only | CON only | Different | Middle | Annual | Experimental | | Arable | Asia | Cfa |
| Heinze et al. 2010 | -0.073 | 0.020 | 590 | 9.5 | 6.6 | 5.0 | 0.86 | 0.080 | ORG only | ORG and CON |  | Similar | Light | Annual | Experimental | Old | Arable | Europe | Cfb |
| Heinze et al. 2010 | -0.150 | 0.020 | 590 | 9.5 | 6.6 | 5.0 | 0.85 | 0.079 | ORG only | ORG and CON |  | Similar |  | Annual | Experimental | Old | Arable | Europe | Cfb |
| Islam and Weil 2000 | -0.198 | 0.021 |  |  | 6.1 | 27.0 | 2.35 | 0.313 | ORG only | ORG and CON |  | Different | Middle | Annual | Experimental | Middle | Arable | North America | Dfa |
| Islam and Weil 2000 | 0.213 | 0.021 |  |  | 6.1 | 27.1 | 2.39 | 0.308 | none | ORG and CON |  | Different | Middle | Annual | Experimental | Middle | Arable | North America | Dfa |
| Jacinthe et al. 2011 | -2.486 | 0.976 | 205 | 20.0 | 7.8 |  | 0.90 |  | ORG only | ORG only |  | Different |  | Annual | Farm | Young | Arable | South America | BSh |
| Jacinthe et al. 2011 | -1.641 | 0.191 | 205 | 20.0 | 7.7 |  | 0.84 |  | ORG only | ORG only |  | Different |  | Annual | Farm | Middle | Arable | South America | BSh |
| Jacinthe et al. 2011 | -1.363 | 0.116 | 205 | 20.0 | 7.8 |  | 0.75 |  | ORG only | ORG only |  | Different |  | Annual | Farm | Middle | Arable | South America | BSh |
| Lagomarsino et al. 2009 | 0.040 | 0.018 |  |  |  |  | 2.10 |  | none | ORG and CON |  | Different |  | Annual | Experimental | Middle | Arable | Europe | Csa |
| Lagomarsino et al. 2009 | -0.987 | 0.186 |  |  |  |  | 1.95 |  | ORG only | ORG and CON |  | Different |  | Annual | Experimental | Middle | Arable | Europe | Csa |
| Lagomarsino et al. 2009 | -0.965 | 0.082 |  |  |  |  | 2.15 |  | ORG only | ORG and CON |  | Different |  | Annual | Experimental | Middle | Arable | Europe | Csa |
| Liu et al. 2007 | 0.775 | 0.046 |  |  |  |  | 1.63 | 0.277 | ORG only | ORG only | CON only | Different |  | Annual | Farm | Old | Arable | North America | Cfa |
| Liu et al. 2007 | 1.088 | 0.073 |  |  |  |  | 1.18 | 0.232 | ORG only | ORG only | CON only | Different |  | Annual | Farm | Old | Arable | North America | Cfa |
| Marinari et al. 2010 | 0.467 | 0.011 |  |  |  |  | 1.19 | 0.135 | ORG only | ORG only | CON only | Similar |  | Annual | Experimental | Middle | Arable | Europe | Csa |
| Mazzoncini et al. 2010 | 0.162 | 0.027 | 700 |  |  |  | 0.87 | 0.105 | ORG only | ORG and CON |  | Similar |  | Annual | Experimental | Middle | Arable | Europe | Csa |
| Melero et al. 2006 | -0.668 | 0.030 |  |  | 8.4 | 23.0 | 1.52 | 0.175 | ORG only | ORG and CON | none | Similar | Middle | Annual | Experimental | Middle | Arable | Europe | Csa |
| Melero et al. 2006 | -0.109 | 0.020 |  |  | 7.8 | 23.0 | 1.46 | 0.155 | ORG only | ORG and CON | none | Similar | Middle | Annual | Experimental | Middle | Orchard | Europe | Csa |
| Migliorini et al. 2014 | 0.606 | 0.028 |  |  | 6.1 | 27.0 | 2.35 | 0.313 | ORG only | ORG and CON |  | Different | Middle | Annual | Experimental | Middle | Arable | Europe | Csa |
| Migliorini et al. 2014 | 0.606 | 0.028 |  |  | 6.1 | 27.1 | 2.39 | 0.308 | none | ORG and CON |  | Different | Middle | Annual | Experimental | Middle | Arable | Europe | Csa |
| Monokrousos et al. 2006 | -0.041 | 0.016 | 506 |  | 7.7 | 26.1 | 1.43 | 0.067 | ORG and CON | none | CON only | Similar | Middle | Annual | Farm | Middle | Arable | Europe | Cfa |
| Monokrousos et al. 2006 | -0.281 | 0.017 | 506 |  | 7.9 | 29.6 | 1.61 | 0.073 | ORG and CON | none | CON only | Similar | Middle | Annual | Farm | Middle | Arable | Europe | Cfa |
| Monokrousos et al. 2006 | -0.080 | 0.016 | 506 |  | 7.9 | 28.2 | 1.42 | 0.068 | ORG and CON | none | CON only | Similar | Middle | Annual | Farm | Young | Arable | Europe | Cfa |
| Oberholzer et al. 2000 | 0.009 | 0.058 |  |  | 6.5 | 26.5 | 1.30 |  | ORG and CON | ORG and CON | no |  | Middle | Annual | Farm | Middle | Arable | Europe | Cfb |
| Protease |  |  |  |  |  |  |  |  |  |  |  |  |  |  |  |  |  |  |  |
| Reganold et al. 2010 | 0.151 | 0.037 |  |  | 7.1 | 13.1 | 0.91 | 0.077 | ORG and CON | ORG and CON | CON only |  | Light | Perennial | Farm | | Orchard | North America | Csb |
| Melero et al. 2006 1 | 0.721 | 0.007 |  |  | 8.4 | 23.0 | 1.52 | 0.175 | ORG only | ORG and CON | none | Similar | Middle | Annual | Experimental | Middle | Arable | Europe | Csa |
| Melero et al. 2006 2 | 0.607 | 0.003 |  |  | 7.8 | 23.0 | 1.46 | 0.155 | ORG only | ORG and CON | none | Similar | Middle | Annual | Experimental | Middle | Orchard | Europe | Csa |
| Marinari et al. 2006 | 0.701 | 0.013 |  |  | 7.6 | 20.0 | 0.96 | 0.100 | ORG only | none | CON only | Similar | Middle | Annual | Farm | Middle | Arable | Europe | Csa |
| Okur et al. 2009 1 | 0.738 | 0.033 | 706 | 17.0 | 7.5 | 15.9 | 0.77 | 0.135 | ORG only | none | CON only | Similar | Light | Perennial | Farm | | Orchard | Asia | Csa |
| Okur et al. 2009 2 | 0.776 | 0.016 | 706 | 17.0 | 7.5 | 15.9 | 0.77 | 0.135 | ORG only | none | CON only | Similar | Light | Perennial | Farm | | Orchard | Asia | Csa |
| Okur et al. 2009 3 | 0.405 | 0.014 | 706 | 17.0 | 7.5 | 15.9 | 0.77 | 0.135 | ORG only | none | CON only | Similar | Light | Perennial | Farm | | Orchard | Asia | Csa |
| Urease |  |  |  |  |  |  |  |  |  |  |  |  |  |  |  |  |  |  |  |
| Bolton Jr et al. 1985 | 0.629 | 0.008 |  |  | 5.8 |  | 1.03 | 0.112 | ORG only | ORG and CON |  | Different |  | Annual | Farm | Old | Arable | North America | BSk |
| Ge et al. 2011 | 0.192 | 0.014 | 1255 | 17.5 | 8.2 | 20.0 | 1.21 | 0.155 | ORG only | none | CON only | Different | Middle | Annual | Experimental | | Arable | Asia | Cfa |
| Ge et al. 2011 | 1.946 | 0.148 | 1255 | 17.5 | 7.8 | 20.0 | 1.75 | 0.252 | ORG only | ORG only | CON only | Different | Middle | Annual | Experimental | | Arable | Asia | Cfa |
| Melero et al. 2006 | 1.424 | 0.429 |  |  | 8.4 | 23.0 | 1.52 | 0.175 | ORG only | ORG and CON | none | Similar | Middle | Annual | Experimental | Middle | Arable | Europe | Csa |
| Melero et al. 2006 | 0.274 | 0.044 |  |  | 7.8 | 23.0 | 1.46 | 0.155 | ORG only | ORG and CON | none | Similar | Middle | Annual | Experimental | Middle | Orchard | Europe | Csa |
| Monokrousos et al. 2006 | 0.449 | 0.002 | 506 |  | 7.7 | 26.1 | 1.43 | 0.067 | ORG and CON | none | CON only | Similar | Middle | Annual | Farm | Middle | Arable | Europe | Cfa |
| Monokrousos et al. 2006 | 0.550 | 0.002 | 506 |  | 7.9 | 29.6 | 1.61 | 0.073 | ORG and CON | none | CON only | Similar | Middle | Annual | Farm | Middle | Arable | Europe | Cfa |
| Monokrousos et al. 2006 | 0.491 | 0.002 | 506 |  | 7.9 | 28.2 | 1.42 | 0.068 | ORG and CON | none | CON only | Similar | Middle | Annual | Farm | Young | Arable | Europe | Cfa |
| Nguyen et al. 1995 | 0.044 | 0.018 | 700 |  | 5.9 |  | 3.40 | 0.295 | ORG only | ORG and CON |  | Different |  | Annual | Farm | Middle | Arable | Oceania | Cfb |
| Nguyen et al. 1995 | -0.005 | 0.004 | 700 |  | 5.7 |  | 3.35 | 0.280 | ORG only | ORG and CON |  | Different |  | Annual | Farm | Middle | Arable | Oceania | Cfb |
| Nguyen et al. 1995 | 0.018 | 0.013 | 700 |  | 6.1 |  | 2.95 | 0.295 | ORG only | ORG and CON |  | Different |  | Annual | Farm | Middle | Arable | Oceania | Cfb |
| Nguyen et al. 1995 | 0.346 | 0.007 | 700 |  | 5.9 |  | 3.70 | 0.330 | ORG only | ORG and CON |  | Different |  | Annual | Farm | Middle | Arable | Oceania | Cfb |
| Nguyen et al. 1995 | -0.019 | 0.010 | 700 |  | 5.8 |  | 3.45 | 0.330 | ORG only | ORG and CON |  | Different |  | Annual | Farm | Middle | Arable | Oceania | Cfb |
| Nguyen et al. 1995 | 0.144 | 0.012 | 700 |  | 6.0 |  | 3.15 | 0.315 | ORG only | ORG and CON |  | Different |  | Annual | Farm | Middle | Arable | Oceania | Cfb |
| Okur et al. 2009 | 0.270 | 0.010 | 706 | 17.0 | 7.5 | 15.9 | 0.77 | 0.135 | ORG only | none | CON only | Similar | Light | Perennial | Farm | | Orchard | Asia | Csa |
| Okur et al. 2009 | 0.251 | 0.013 | 706 | 17.0 | 7.5 | 15.9 | 0.77 | 0.135 | ORG only | none | CON only | Similar | Light | Perennial | Farm | | Orchard | Asia | Csa |
| Okur et al. 2009 | 0.277 | 0.011 | 706 | 17.0 | 7.5 | 15.9 | 0.77 | 0.135 | ORG only | none | CON only | Similar | Light | Perennial | Farm | | Orchard | Asia | Csa |
| Velmourougane 2016 | -0.292 | 0.010 | 1700 |  | 5.4 | 25.0 | 2.00 | 0.315 | ORG only | none | CON only | Different | Middle | Perennial | Experimental | Old | Orchard | Asia | Am |
